# DSF-YOLO: Multi-Scale Fire and Smoke Detection Based on YOLO11

**DOI:** 10.3390/s26206506

**Published:** 2026-10-14

**Authors:** Chengzhi Ren, Hongyan Ma, Yuchen Zhang, Wenwen Yu

**Affiliations:** School of Intelligent Science and Technology, Beijing University of Civil Engineering and Architecture, Beijing 100044, China

**Keywords:** fire and smoke detection, YOLO11, wavelet processing, feature fusion, bounding-box regression

## Abstract

Detecting fire and smoke across a wide range of object sizes requires both spatial detail and contextual information. However, incorporating these features into a compact detector remains a challenge. This study presents DSF-YOLO, a fire and smoke detector based on YOLO11n. C3K2-MFB introduces wavelet processing into C3K2 to represent coarse structures and fine details in separate frequency subbands. DyFusion Block combines local convolutional features with transformer-based context to support complementary feature processing. ADown is also introduced to reduce the cost of downsampling. For bounding-box regression, AIM-MSAIoU combines auxiliary-box overlap at a fixed scale of 0.7 with a shape-weighted corner penalty normalized by target size. The model is evaluated on the Dual-Scale Fire (DSF) dataset, which contains 7572 images and four scale-based fire and smoke classes. Compared with YOLO11n, DSF-YOLO increases *mAP*@0.5 from 0.905 to 0.919 and *mAP*@[0.5:0.95] from 0.631 to 0.680. At the same time, parameter count decreases by 8.14% to 2.37 M, and *FLOPs* decrease by 4.76% to 6.0 G *FLOPs*. Reported throughput increases from 164.0 to 175.0 FPS on an RTX 4090D at a batch size of 16. The results show that DSF-YOLO improves detection accuracy while reducing computational cost under the reported conditions.

## 1. Introduction

Early detection of fire and smoke can support a timely response to threats to life and property [1]. Advances in imaging and deep learning have therefore encouraged research into vision-based fire monitoring [1,2]. Object detectors commonly follow either a two-stage or a one-stage design. Two-stage detectors, such as Faster R-CNN [3], generate candidate regions before classifying them and refining their bounding boxes. In contrast, one-stage detectors, such as YOLO [4], predict object classes and locations in a single forward pass. This unified prediction process makes one-stage detection relevant to continuous monitoring, where both detection accuracy and processing speed matter.

YOLO established a unified, single-pass prediction of object locations and classes [4]. Later versions introduced multi-scale prediction and feature aggregation for objects of different sizes: YOLOv3 predicts at several feature-map resolutions [5], and YOLOv4 and YOLOv5 further develop extraction and aggregation strategies [6,7]. Feature fusion has advanced in parallel, from the top-down pathway of Feature Pyramid Networks [8] and the bottom-up path of Path Aggregation Network [9] to the weighted bidirectional fusion of EfficientDet [10]. Recent detectors explore different forms of feature interaction, including attention-centric YOLOv12 [11] and hypergraph-based YOLOv13 [12]; the compact YOLO26n [13] is also included in this study as a comparison model. These architectures provide a range of reference designs for assessing the accuracy and computational cost of task-specific modifications.

Despite these advances, fire and smoke introduce challenges that are not fully described by object size alone. Flame appearance changes across combustion stages, and smoke may have diffuse boundaries, low contrast and partial transparency. Viewing distance also produces large-scale differences, from small, distant flames to broad smoke plumes [1,14,15,16]. Recent studies address these difficulties through both feature design and training supervision. YOLO-Fire adds a P2 detection layer and coordinate attention for multi-scale fire detection [17,18]. SFFA-YOLO combines cross-scale feature fusion, channel–spatial feature processing and dynamic supervision for fire and smoke targets [19]. Other studies investigate compact convolutional networks and attention-based feature processing to balance recognition performance and computational cost [20,21,22,23,24]. These approaches provide useful design directions, but small smoke regions remain difficult to distinguish from clouds, haze and other visually similar objects [16,25]. Thus, increasing feature resolution alone may be insufficient: the detector also needs contextual information to interpret weak local evidence. This requirement is particularly relevant to continuous monitoring, where additional feature processing must be considered alongside inference cost.

These observations motivate a detector that combines spatial detail, broader context and suitable localization supervision within a compact architecture. Multi-scale fusion can bring low-level localization cues together with high-level semantic information [9,10], but its benefit depends on how features are processed and how much computation is added. We therefore develop DSF-YOLO from YOLO11n, with coordinated changes to feature extraction, feature interaction, downsampling and bounding-box regression. Wavelet processing provides separate representations of coarse structures and detail; local and Transformer branches combine complementary information; ADown limits downsampling cost; the revised regression objective supervises box overlap and corner alignment. The contributions are summarized as follows:

(1) C3K2-MFB introduces wavelet processing into C3K2, using frequency subbands to represent coarse spatial structures and fine details within the feature extraction stage.
(2) DyFusion Block combines a local convolutional branch with a Transformer branch, allowing local features and broader contextual information to be processed within one block.
(3) AIM-MSAIoU combines fixed-scale auxiliary-box overlap with a shape-weighted corner penalty normalized by target size, providing complementary constraints for bounding-box regression.

Section 1 introduces the background and motivation of this study. Section 2 describes the model architecture and its components. Section 3 presents the datasets, experimental settings, comparative results and ablation analysis. Section 4 discusses the conclusions and remaining limitations.

## 2. Proposed Method

YOLO11 is a one-stage detector whose architecture includes C3k2 feature-extraction modules and C2PSA for contextual feature interaction [26]. Its backbone extracts features at successive spatial resolutions, while the neck combines information for detection at multiple scales. We use YOLO11n as the baseline because these components provide suitable locations for introducing frequency processing, contextual modeling and more economical downsampling. This common starting point allows each modification to be examined through matched ablation configurations. The complete model is compared with lightweight YOLO variants, including YOLOv12 and YOLOv13 [11,12], under the reported experimental settings.

Fire and smoke detection present several related challenges: weak or blurred local boundaries, background patterns with similar appearances, and large differences in object size. Repeated downsampling can also remove spatial information needed to localize small objects. Together, these challenges motivate complementary changes in feature representation and box supervision: local detail and scene context support recognition, while the downsampling strategy and regression objective influence the information available for localization.

**Figure 1:**
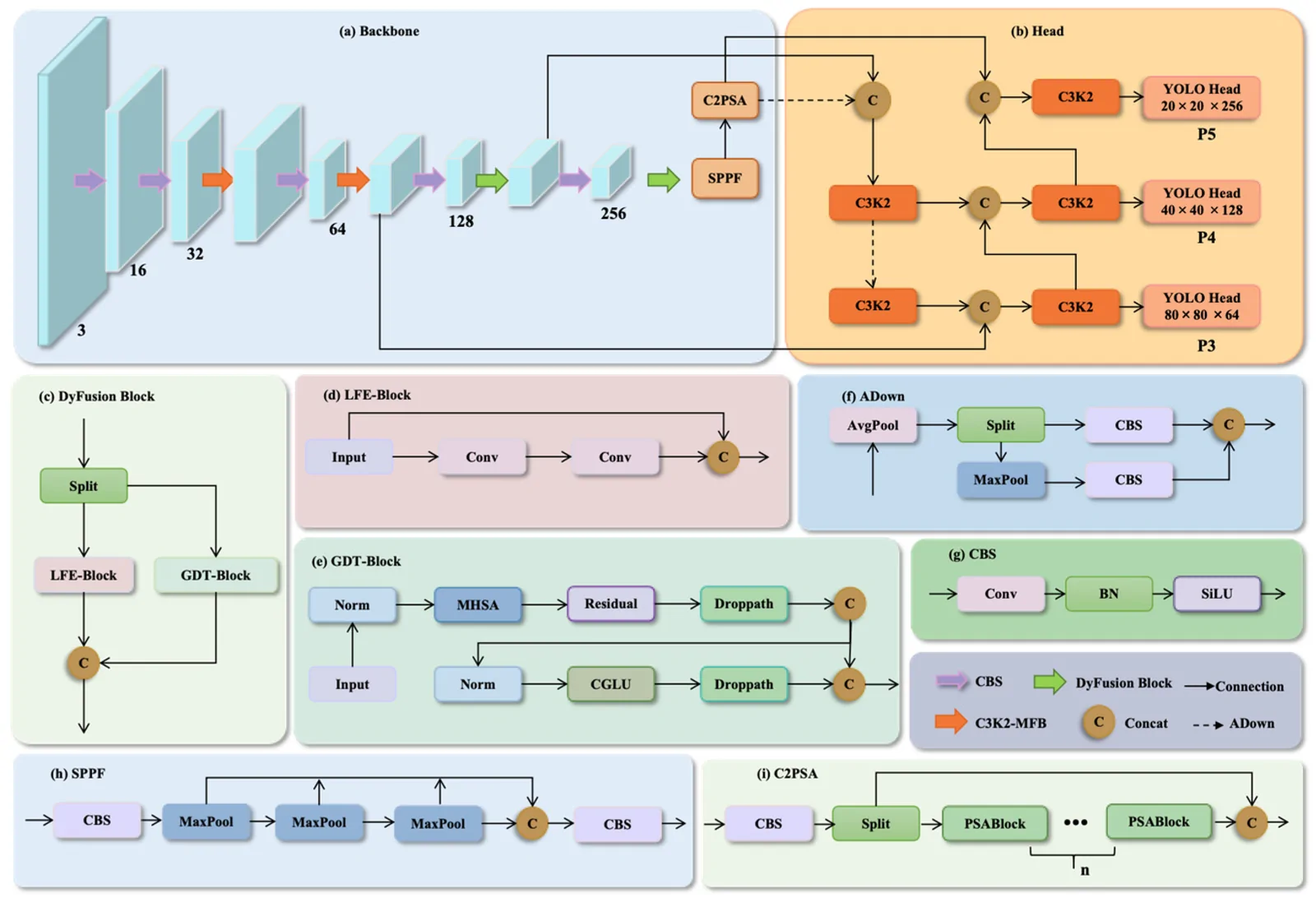
Overall architecture of DSF-YOLO: (**a**) backbone, in which C3K2-MFB (orange) and DyFusion Block (green) mark the modified stages; (**b**) head, with detection heads at the P3, P4 and P5 levels and ADown shown as dashed paths; (**c**) DyFusion Block; (**d**) LFE-Block; (**e**) GDT-Block, combining an MHSA path and a CGLU path through residual addition and DropPath; (**f**) ADown; (**g**) CBS; (**h**) SPPF; and (**i**) C2PSA. C denotes channel concatenation. P3–P5 denotes feature-pyramid levels; channel widths are schematic.

To address these challenges, DSF-YOLO introduces four changes to the YOLO11n framework (Figure 1). C3K2-MFB combines wavelet decomposition with learnable filtering and channel weighting to represent coarse structures and local detail. DyFusion Block then complements local convolutional processing with self-attention, allowing features from different positions to interact. ADown replaces selected downsampling operations with a two-branch channel structure to reduce convolutional cost. Finally, AIM-MSAIoU modifies box-regression supervision using auxiliary-box overlap and shape-weighted corner distances. The first three changes affect feature processing, whereas the loss acts during training. Their separate and combined effects are evaluated in the ablation study.

### 2.1. C3K2-MFB

Fire and smoke may contain both broad, slowly varying regions and fine boundary or texture changes. A single spatial scale may, therefore, provide an incomplete description of their appearance. C3K2-MFB introduces wavelet processing into C3K2 to represent these components at multiple resolutions. Wavelet decomposition separates an approximation component from directional detail components [27], while learnable filtering and inverse reconstruction follow the wavelet-convolution principle [28]. SE subsequently adjusts channel responses according to the input [29]. Input connections allow frequency-processed information to be combined with spatial features. The resulting representation is intended to retain structural cues at different scales for subsequent detection.

**Figure 2:**
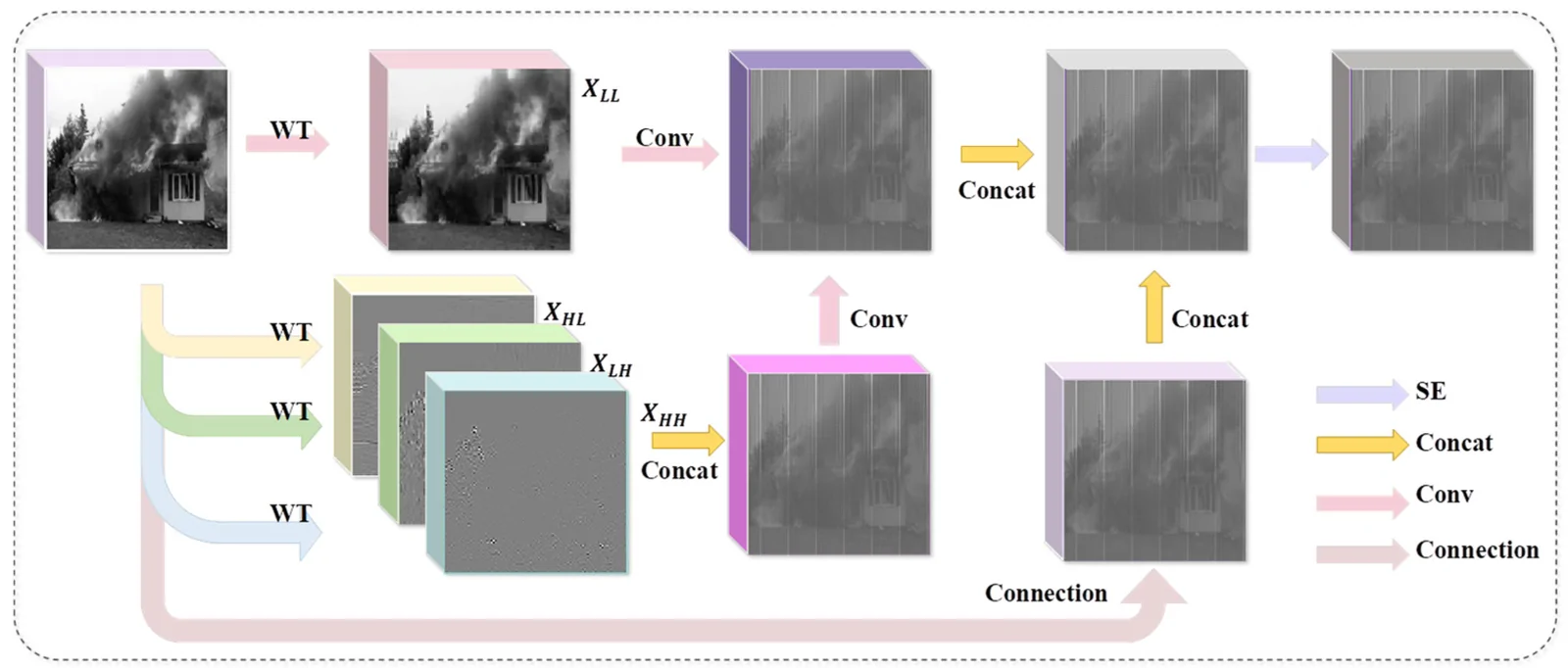
Conceptual frequency-processing path in the MFB bottleneck. Approximation and detail bands undergo learnable filtering and inverse reconstruction before feature combination and channel recalibration.

**Figure 3:**
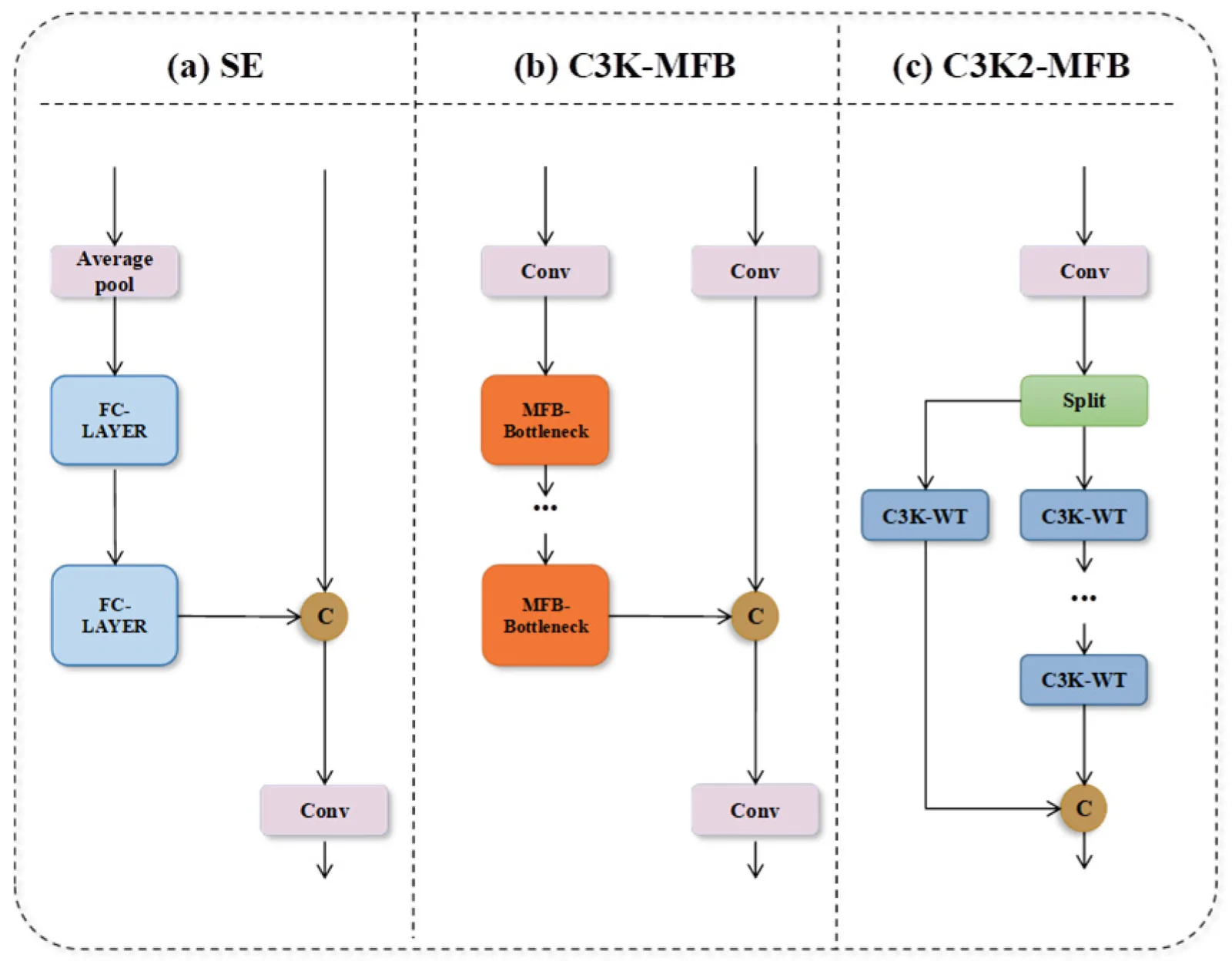
Component schematics: (**a**) SE channel weighting; (**b**) C3K-MFB; and (**c**) C3K2-MFB. SE multiplies channels by learned weights; the C3K branches use feature concatenation. Repetition counts and channel widths are omitted.

Figure 2 and Figure 3 show the frequency-processing path and its integration into C3K2-MFB. A two-dimensional wavelet transform produces an approximation band, *LL*, and three detail bands: *LH*, *HL* and *HH*. *LL* represents coarse spatial variation, whereas the detail bands describe changes along different directions; these bands do not correspond directly to object categories. For multilevel analysis, the *LL* component is decomposed again, providing access to progressively coarser structure. In Equation (1), Conv denotes fixed wavelet filtering with downsampling, not the learnable convolution subsequently applied to the sub-bands. This distinction separates the decomposition operation from feature learning:

[*X_LL_*,*X_LH_*,*X_HL_*,*X_HH_*] = *Conv*([*f_LL_*,*f_LH_*,*f_HL_*,*f_HH_*],*X*) (1)

The filters *f*_LH_, *f*_HL_, *f*_HH_ and *f*_LL_ generate the four sub-bands. Equation (2) describes recursive decomposition, initialized with $X_{\mathrm{LL}}^{\left(0\right)}=X$. Equation (3) illustrates a single level of wavelet-domain filtering with learnable weights W followed by inverse reconstruction. With multiple decomposition levels, reconstruction proceeds from coarse to fine [28]; Equation (3) alone is therefore not a complete specification of a multilevel implementation.

(2) $$\left\{X_{\mathrm{LL}}^{\left(i\right)},X_{\mathrm{LH}}^{\left(i\right)},X_{\mathrm{HL}}^{\left(i\right)},X_{\mathrm{HH}}^{\left(i\right)}\}=\mathrm{WT}(X_{\mathrm{LL}}^{\left(i-1\right)}\right)$$

*Y* = *IWT*(*Conv*(*W* ⋅ *WT*(*X*))) (3)

SE first applies global average pooling to obtain one descriptor per channel. Two fully connected layers then reduce the descriptor dimension from C to C/r and restore it to C, with a nonlinear activation between the layers and a final sigmoid. The resulting weights multiply the corresponding feature channels [29]. For example, C = 256 and r = 16 give a 16-dimensional hidden representation; this example does not prescribe the channel dimensions at every stage. Within C3K2-MFB, channel weighting complements wavelet processing by adjusting the contributions of the reconstructed features. The combined module is used in backbone feature extraction.

### 2.2. DyFusion Block

Frequency processing can describe local variation, but local appearance alone may remain ambiguous when background regions resemble fire or smoke. Relating a candidate region to its surroundings provides an additional source of information [14,15,16,21,22]. This motivates the contextual branch introduced in DyFusion Block.

**Figure 4:**
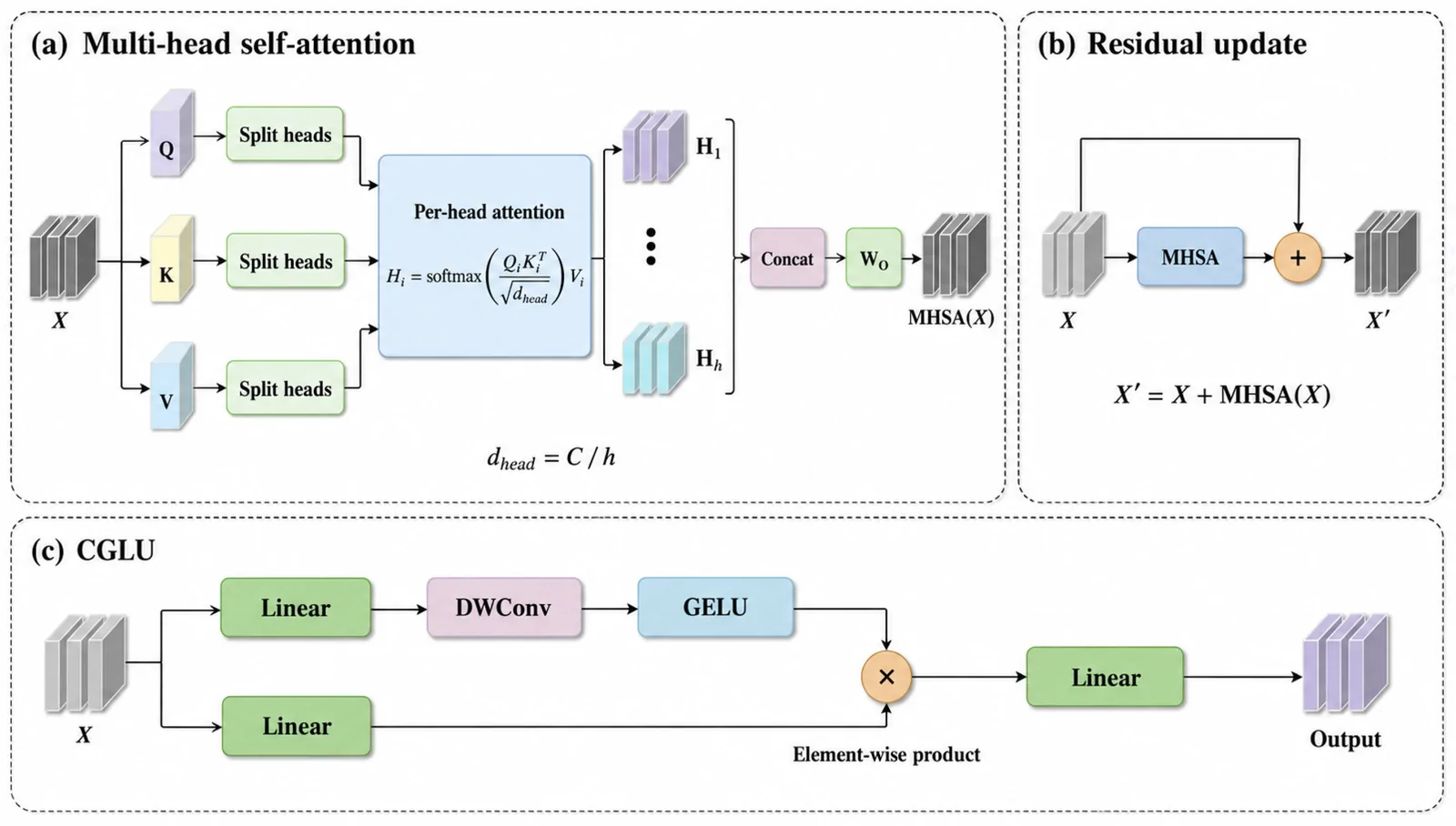
GDT-Block principles: (**a**) multi-head self-attention; (**b**) residual addition; and (**c**) convolutional GLU [30,33]. Normalization and training-time DropPath are omitted in this schematic.

Self-attention models dependencies between feature positions [30,31]. Hierarchical visual Transformers also demonstrate ways of organizing contextual processing while controlling its cost [32], although the cost depends on the attention design and input resolution. Following this motivation, DyFusion Block combines a Local Feature Extraction Block (LFE-Block) with a Global Dependency Transformer Block (GDT-Block) (Figure 4). The input features are split into two branches, processed separately and then combined. The local branch captures nearby spatial patterns, while the attention branch enables interaction across positions. Combining these branches is intended to retain local evidence and use scene context to help distinguish target regions from similar background appearances.

LFE-Block applies two 3 × 3 convolutions and combines their output with the input features. The input connection carries intermediate features alongside the convolutional path. GDT-Block contains multi-head self-attention (MHSA) followed by a convolutional gated linear unit (CGLU). In GDT-Block, normalized features are projected into queries Q, keys K and values V. Following the multi-head attention formulation [30], these projections are partitioned into h heads. Within each head, query–key similarities determine the attention weights, which are applied to the values. The scaled dot-product operation is:

(4) $${\mathrm{Attention}}_{i}=\mathrm{Softmax}(Q_{i}K_{i}^{T}/\sqrt{d_{\mathrm{head}}})\cdot V_{i}$$

*d_head_* = *c*/*h* is the feature dimension of each head, with C divisible by h. The outputs of all heads are concatenated and mapped by the output projection W_O_:

*MultiHead*(*Q*,*K*,*V*) = *Concat*(*Attention*_1_,…,*Attention_h_*)*W_O_* (5)

The projected attention output is combined with the input through a residual connection. DropPath denotes stochastic depth applied to the attention branch during training. The addition in Equation (6) combines the input representation with attention-derived information; reshaping between feature maps and token sequences is a separate operation:

*X_out_* = *X* + *DropPath*(*MultiHead*(*Q*,*K*,*V*)) (6)

The residual output therefore contains value features weighted by dependencies across positions. CGLU complements this interaction with local processing and multiplicative gating. In the cited CGLU formulation, one projected branch passes through a depthwise convolution and GELU before multiplying the other branch; an output projection follows [33]. This introduces local spatial information into the gate, complementing the interactions modeled by self-attention. DyFusion is used in deeper feature processing, where lower spatial resolution helps limit the cost of attention. Its contribution to detection is assessed through the corresponding ablations.

### 2.3. ADown

Feature enrichment also requires attention to computational cost. As shown by the module-level ablation (Section 3.4.1), the standalone DyFusion and C3K2-MFB variants have slightly fewer parameters and *FLOPs* than YOLO11n, but their reported throughput falls from 164.0 FPS to 141.7 and 104.5 FPS, respectively. These values correspond to throughput reductions of 13.6% and 36.3%. We therefore adopt ADown from YOLOv9 to reduce the convolutional cost of selected downsampling operations [34].

**Figure 5:**
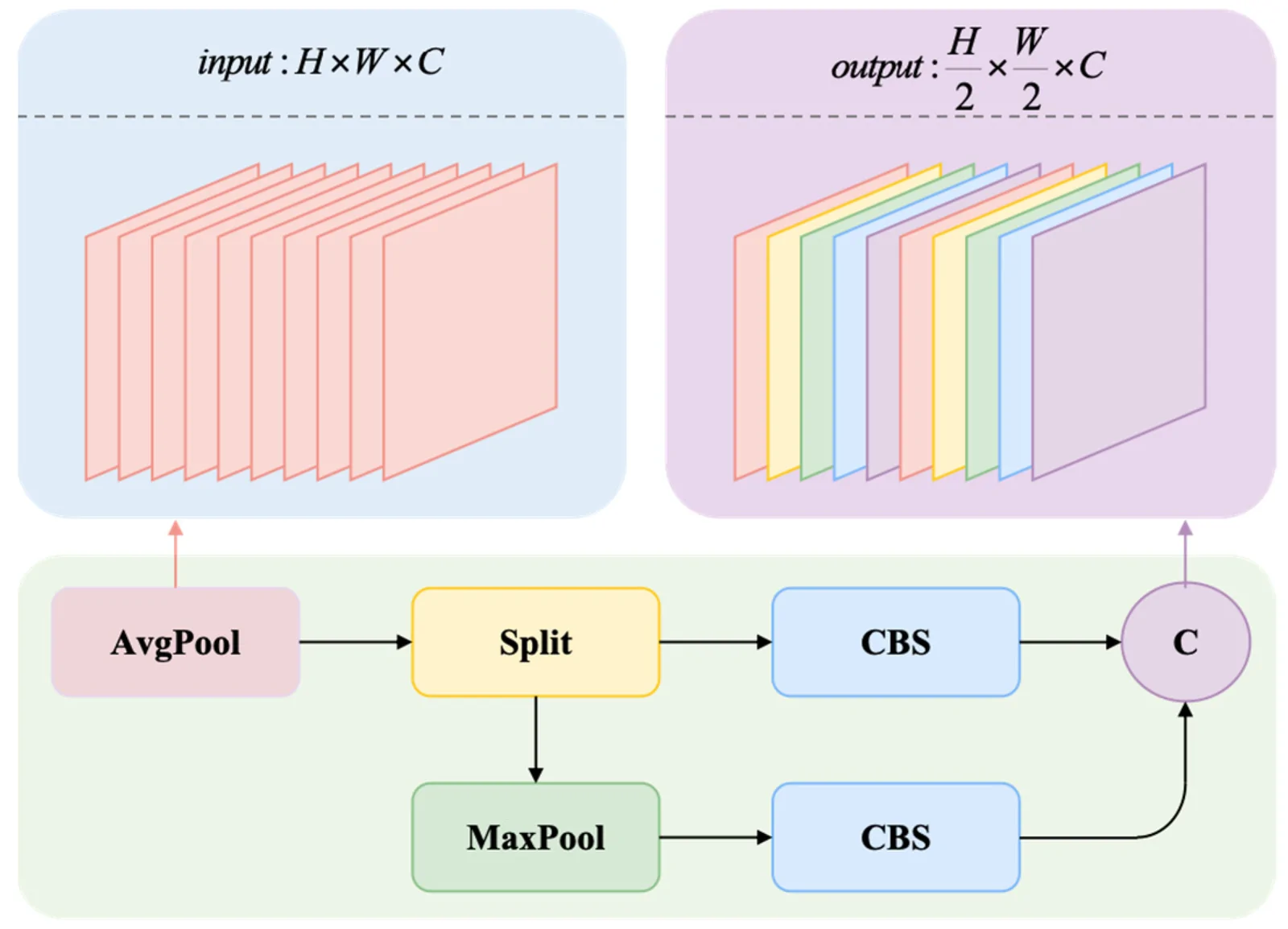
ADown operations [34]. Each branch processes half of the input channels and produces half of the output channels. CBS denotes convolution, batch normalization and SiLU activation.

Given an input of size *H* × *W* × *C_in_* and even input and output channel counts, ADown first applies 2 × 2 average pooling with stride 1 and no padding (Figure 5). The result is divided equally along the channel dimension. One branch uses a 3 × 3 convolution with stride 2 and padding 1. The other uses 3 × 3 max pooling with stride 2 and padding 1, followed by a 1 × 1 convolution. The convolutions include batch normalization and SiLU activation. Concatenating the branch outputs gives C_out_ channels and, for even H and W, spatial dimensions *H*/2 × *W*/2. The configured C_out_ need not equal C_in_.

Compared with applying a full-channel 3 × 3 stride-2 convolution, ADown can lower convolutional cost because each branch processes only half of the input channels and the pooling branch uses a 1 × 1 convolution [34]. The branches also apply different spatial transformations: one learns a strided filter, while the other selects local maxima before channel mixing. Their outputs are combined to retain complementary information, although downsampling still reduces spatial resolution. In DSF-YOLO, ADown replaces the P3–P4 and P4–P5 downsampling transitions. Its effect on accuracy and throughput depends on the surrounding modules and is therefore examined for each ablation configuration, rather than inferred from *FLOPs* alone.

### 2.4. AIM-MSAIoU

**Figure 6:**
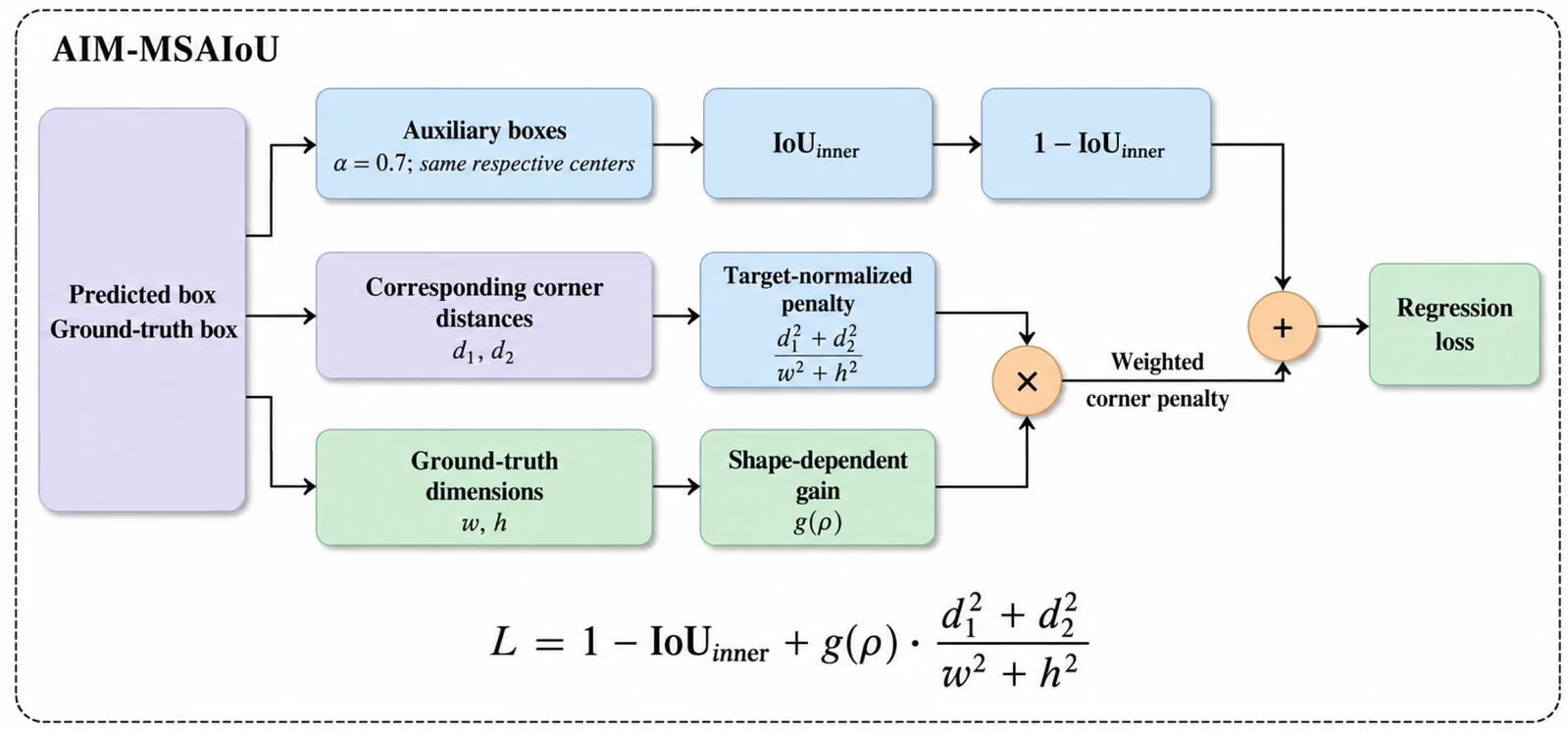
AIM-MSAIoU components in Equations (7) and (8). The loss combines one minus auxiliary-box IoU with a shape-weighted, target-normalized corner penalty; it contains no separate angular-cost term.

Bounding-box regression measures geometric agreement between predicted and ground-truth rectangles. IoU-based extensions introduce additional signals for different overlap conditions [35]. CIoU combines overlap, center distance and aspect ratio [36]. However, when two boxes share an aspect ratio, its ratio term does not directly measure differences in their absolute widths and heights; the overlap and center-distance terms still provide supervision. This motivates using corresponding corners to describe residual position and size errors. For fire and smoke, whose annotated extents may be difficult to localize, AIM-MSAIoU combines this corner supervision with auxiliary-box overlap. It remains a loss for axis-aligned bounding boxes and does not directly represent irregular object contours. Figure 6 summarizes its components.

**Figure 7:**
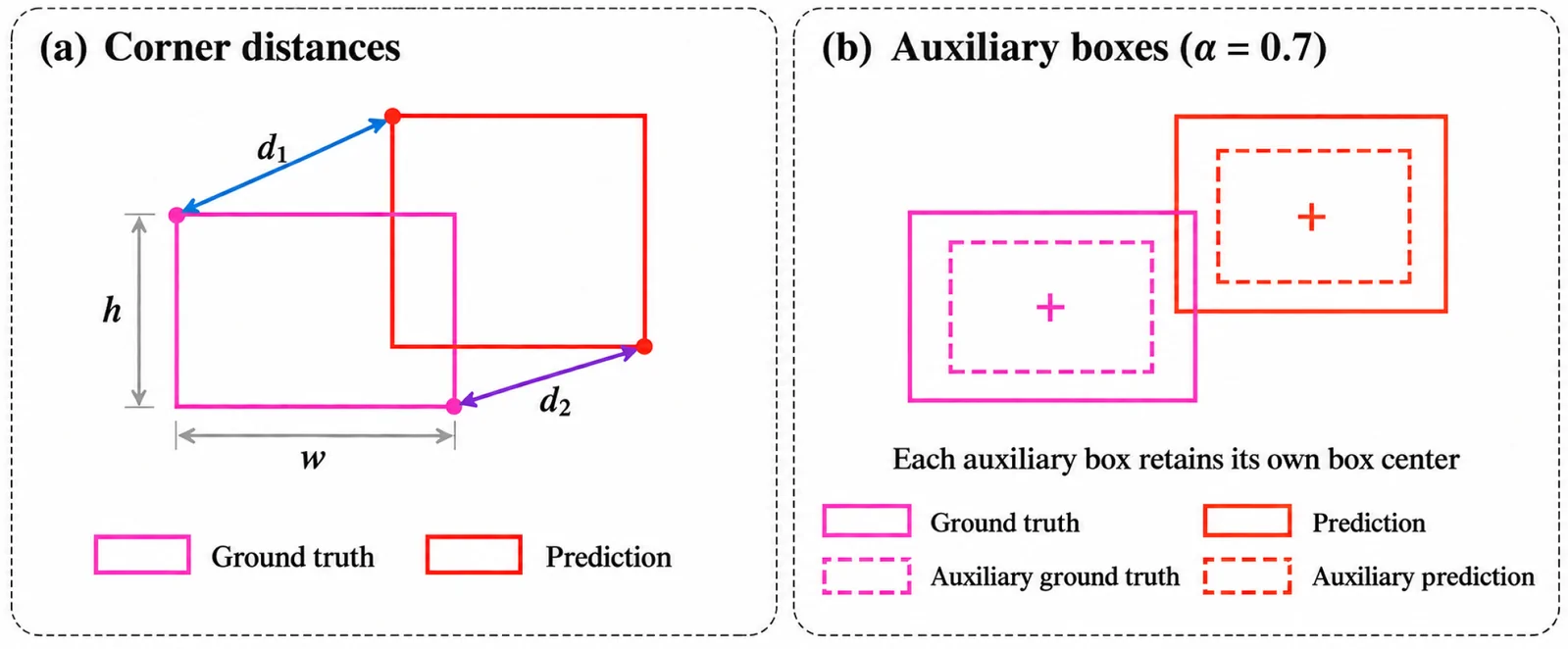
Loss geometry: (**a**) corresponding top-left and bottom-right corner distances, with ground-truth width w and height h; and (**b**) auxiliary boxes scaled about their respective centers by *α* = 0.7.

AIM-MSAIoU combines the corner-distance principle of MPDIoU [37] with the auxiliary-box strategy of Inner-IoU [38]. In this manuscript, MSAIoU denotes the corner-based variant normalized by target dimensions. The top-left and bottom-right corners jointly determine an axis-aligned box, so their distances provide supervision for both position and size errors (Figure 7a). This use of corresponding corners differs from explicitly penalizing only the distance between box centers.

For the overlap component, each predicted and ground-truth box is scaled about its own center, with its width and height multiplied by the fixed ratio α = 0.7 (Figure 7b). The resulting auxiliary boxes are used to compute IoU_inner. Shrinking the boxes can increase sensitivity when their overlap is already high, but also reduces the range over which they overlap [38]. The fixed ratio therefore controls a trade-off between the effective overlap range and sensitivity to the alignment of already overlapping boxes. The loss is defined as:

(7) $$L_{\mathrm{AIM}-\mathrm{MSAIoU}}=1-{\mathrm{IoU}}_{\mathrm{inner}}+\left[a_{1}\sigma (\rho )+\left(1-\sigma (\rho )\right)a_{2}\right]\cdot \frac{d_{1}^{2}+d_{2}^{2}}{w^{2}+h^{2}}$$

The logistic mixing function is defined as:

(8) $$\sigma (\rho )=\frac{1}{1+\exp(-k(\rho -\tau ))}$$

d_1_ and d_2_ are the distances between corresponding top-left and bottom-right corners. The positive quantities w and h are the ground-truth width and height. MPDIoU normalizes corner distances by the squared input-image diagonal [37], whereas Equation (7) uses w^2^ + h^2^ for the target. The penalty is consequently relative to target size and can become large for small boxes; the loss is not restricted to [0, 1].

The shape ratio *ρ* = |*w* − *h*|/*max*(*w*,*h*) lies in [0,1), equals zero for square boxes and approaches one as boxes become increasingly elongated. The gain *g*(*ρ*) = *a*_1_*σ*(*ρ*) + *a*_2_[1 − *σ*(*ρ*)] mixes two coefficients. For a non-negative corner penalty, g must remain non-negative over the admissible range; *a*_1_,*a*_2_ ≥ 0 are sufficient conditions. When *k* > 0, σ increases with ρ, so elongated targets receive greater gain only if *a*_1_ > *a*_2_. The parameters τ and k determine the transition location and slope. Training minimizes LAIM-MSAIoU in place of the CIoU term, while retaining the classification and distribution focal loss terms.

The resulting loss links auxiliary overlap to corner alignment and weights the corner error according to target geometry. Its adaptive element is this shape-dependent gain; the auxiliary-box ratio remains fixed, and no separate angular term is used. We evaluate this regression objective through the controlled loss comparison (Section 3.4.2) and the matched ablation configurations (Section 3.4.1).

## 3. Experimental Results and Analysis

### 3.1. Datasets

A dataset with explicit size labels was needed to evaluate fire and smoke detection across object scales. Public resources such as D-Fire [39], FASDD [40] and FLAME [41] provide imagery from different settings, but their labels and evaluation protocols should not be treated as interchangeable. We therefore constructed the Dual-Scale Fire (DSF) dataset using publicly available imagery and the Wildfire Smoke dataset released by AI for Mankind in collaboration with HPWREN [42]. Targets were outlined in LabelMe, and the polygons were converted to axis-aligned bounding boxes. Every image was named after its source and image index at collection time, so that the provenance of each image remains traceable throughout dataset construction. A second annotator checked all boxes, corrupted and near-duplicate images were removed, and a perceptual-hash comparison across sources was applied to exclude near-duplicates shared between different collections. To quantify annotation consistency, the second annotator independently re-annotated 250 images sampled proportionally from the four classes; 92.3% of the re-annotated boxes matched the released boxes at an IoU of at least 0.5, and the residual disagreements were concentrated on the outer boundaries of transparent smoke plumes.

An instance was defined as small when its bounding box occupied less than 1% of the area of the letterboxed 640 × 640 input, and combining this size rule with the fire and smoke categories produced four classes: large fire (LF), large smoke (LS), small fire (SF) and small smoke (SS). The 1% threshold was fixed before training by inspecting the distribution of annotated box-area ratios, which is bimodal and separates small from large instances in the vicinity of 1%; the threshold therefore lies at the natural boundary between the two modes rather than at a value selected for its effect on the reported results. For reference, 1% of the 640 × 640 evaluation input corresponds to a box area of 4096 pixels, which lies between the small and medium boundaries of the COCO area bands.

**Figure 8:**
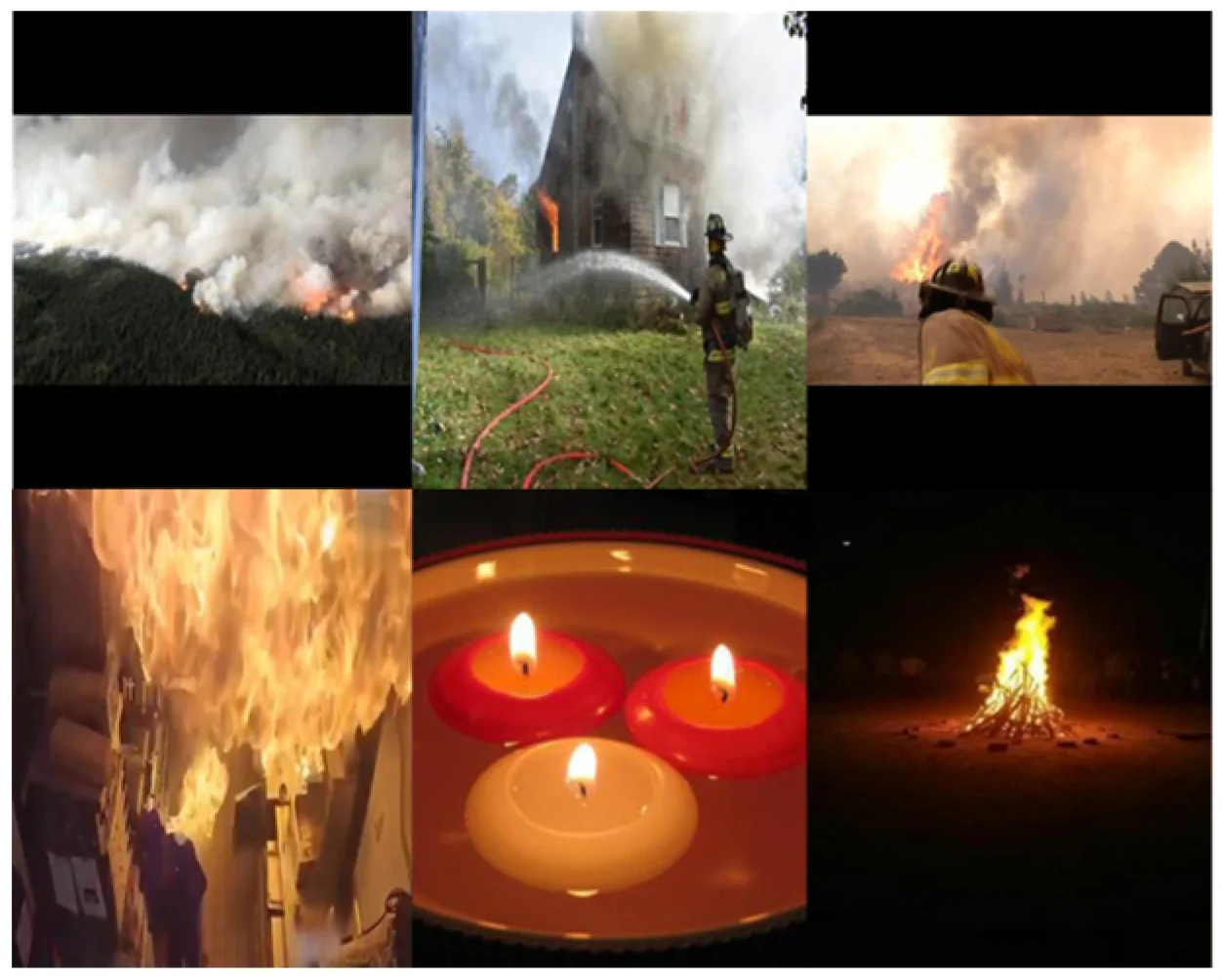
DSF dataset sample figures.

The resulting 7572 images were divided by source-level grouped splitting into training, validation and test subsets at an 8:1:1 ratio with a fixed seed: images sharing an HPWREN camera sequence, fire event, or originating dataset were assigned to a single subset, so that the training and test scenes are independent. The split lists and source-identifier manifest are available from the corresponding author upon reasonable request, and the previous random split at the same ratio is retained in Appendix A for comparison. Table 1 summarizes the number of images and instances of each class in each subset, and confirms that all four classes retain a sufficient number of instances in the validation and test subsets. This split was used throughout the DSF experiments. Figure 8 presents representative samples.

Table 2 further examines the consequences of the class definition. Re-evaluating the class labels at thresholds of 0.5% and 2% changes *mAP*@0.5 by at most 0.2 percentage points and *mAP*@[0.5:0.95] by at most 0.5 points relative to the adopted threshold, indicating that the reported accuracy is insensitive to this choice. The instance counts vary monotonically with the threshold, as expected from the set-inclusion relation between the corresponding small-object sets. Table 3 reports size-stratified *AP* computed with the COCO area bands, an assessment independent of the class definition: DSF-YOLO improves over YOLO11n across all size bands, with the largest margin on small objects, as analyzed in Section 3.3.1. Because size is part of the class label, the resulting *mAP* evaluates a four-class detection task and should not be interpreted as conventional two-class fire and smoke accuracy or as an independently computed small-object *AP*; merged two-class results are reported in Section 3.3.1, and the native two-class benchmark results are reported in Section 3.3.2.

**Figure 9:**
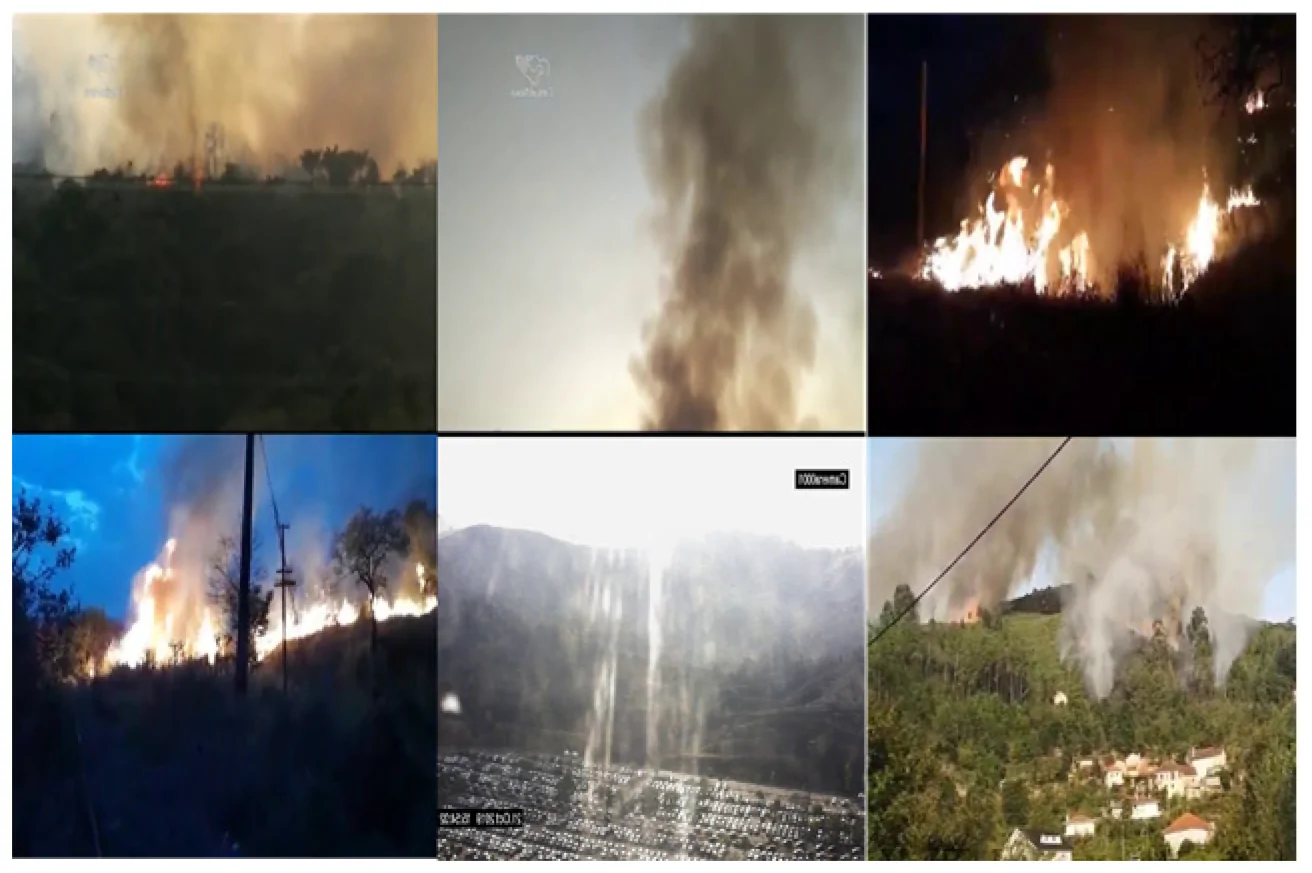
D-Fire dataset sample figures.

To assess generalization within the fire and smoke domain, an additional benchmark evaluation was further conducted on D-Fire [39], an open-access benchmark curated specifically for fire and smoke detection: all models were retrained on its official training set and evaluated on its official test set, so that no DSF imagery is involved in this evaluation (Section 3.3.2). D-Fire contains 21,527 images labeled with the conventional two-class fire and smoke taxonomy (Figure 9), in contrast to the four scale-based classes of DSF; approximately 46% of its images are background scenes containing neither fire nor smoke, and false alarms on these images are included in the evaluation as part of the measured domain gap. The official training, validation, and test split are adopted without modification. For comparability, all models are trained and evaluated under the native two-class taxonomy of D-Fire; the protocols and results are detailed in Section 3.3.2.

**Figure 10:**
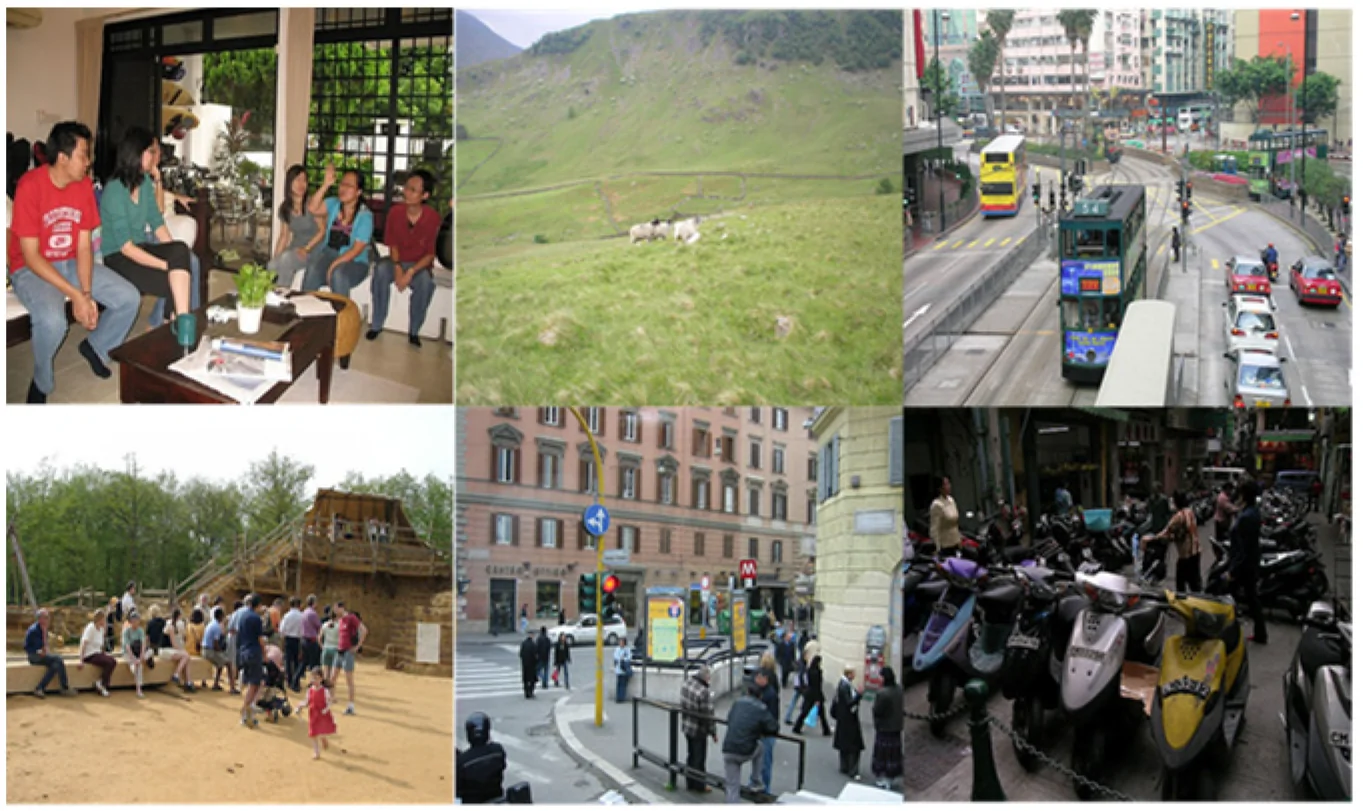
Example images from PASCAL VOC2012 and VisDrone2019.

To examine whether the proposed changes remain beneficial beyond the fire and smoke domain, additional comparisons were conducted on VOC2012 and VisDrone2019, as shown in Figure 10. These two benchmarks are not fire-specific: VOC2012, which includes 20 categories and 11,540 annotated train/val images that were re-split into training, validation and test subsets at an 8:1:1 ratio, evaluates generic multi-scale object detection, and VisDrone2019, comprising 6471 training and 548 validation images captured from diverse environments, evaluates small-object detection in aerial imagery. They therefore serve as a reference for transferable feature learning rather than as evidence of generalization across fire and smoke conditions.

### 3.2. Experimental Environment and Evaluation Indicators

Table 4 summarizes the reported experimental environment. Models were trained for 150 epochs using a batch size of 16, an initial learning rate of 0.01 and an input resolution of 640 × 640 pixels. Hyperparameters were fixed across all compared methods. They were set on the initial split and were not retuned after the source-grouped re-splitting described in Section 3.1, in order to avoid tuning leakage into the test set. All speed values in this manuscript follow a single timing configuration: timing covers the full inference pipeline, namely letterbox preprocessing, the forward pass and NMS post-processing, while image loading and host-to-device transfer are excluded; inputs are 640 × 640 letterboxed images from the corresponding test set. Measurements were taken with one script in one session on the same RTX 4090D under FP32 precision, using 50 warm-up iterations followed by 200 timed iterations with torch.cuda.synchronize() before and after timing. Every model and ablation configuration in this work was profiled under this protocol, so the same configuration reports the same FPS wherever it appears. For the configurations trained with three random seeds, the FPS entries in Table 5 and in the module-level ablation (Section 3.4.1) are the mean ± standard deviation over timing runs of the three seed checkpoints; for the remaining configurations, FPS is the mean of a single timing run. Table 5 also lists batch-1 latency in milliseconds per image for real-time monitoring relevance, and the remaining tables report mean FPS. *FLOPs* count multiply–accumulate operations of the forward pass only and do not capture memory traffic or kernel-launch overhead; configurations with similar *FLOPs* can therefore differ in measured throughput, which is analyzed at the operator level in Appendix C.

To show the effectiveness of the proposed algorithm improvements, the selected evaluation metrics include precision (P), recall (R), mean average precision (*mAP*), number of parameters (Params), Model Size, floating-point operations (*FLOPs*), and frames per second (FPS). Precision and recall are reported at a fixed confidence threshold of 0.25 rather than at the maximum-F1 operating point; this choice is stated for reproducibility. Average Precision (*AP*) denotes the average precision across different recall conditions, numerically corresponding to the integral of the area under the P-R curve, and represents the precision of correctly predicting a certain class of target. *mAP* is the mean of *AP* values over all predicted classes, computed as in Equation (9), reflecting the overall detection performance of the model. Specifically, *mAP*@0.5 denotes the average precision when the Intersection-over-Union (IoU) threshold is set at 0.5, while *mAP*@[0.5:0.95] refers to the mean average precision across IoU thresholds incrementally varying from 0.5 to 0.95 in increments of 0.05. Higher *mAP* values indicate better overall model accuracy.

(9) $$\mathrm{mAP}=\frac{1}{N}\sum_{i=1}^{N}AP_{i}$$

These cost measures describe different aspects of a detector. Parameter count quantifies the number of learned values, and model size refers to storage occupied by the saved weights. Neither directly measures peak runtime memory, which also includes intermediate activations and other buffers. FPS measures throughput and also depends on hardware, software, numerical precision, batching and the processing stages included in timing. Consequently, a reduction in parameters or *FLOPs* does not by itself guarantee a proportional speed increase.

The key configurations, namely the YOLO11n baseline, the four single-module variants, D + W, A + D + W, and the complete DSF-YOLO, were each trained with three random seeds; each corresponding row reports the mean followed by the sample standard deviation. Standard deviations are computed with an *n* − 1 denominator. The remaining configurations use single runs under the identical protocol, as noted in the table captions. Component-level differences smaller than one standard deviation are discussed as cost-side effects rather than claimed as accuracy improvements.

### 3.3. Comparative Experiments

#### 3.3.1. Comparison Experiment on the DSF Dataset

Table 5 compares DSF-YOLO with conventional detectors, lightweight alternatives and two fire- and smoke-specific detectors under a common evaluation and timing protocol. Relative to Faster R-CNN and SSD, DSF-YOLO reduces parameter count by 91.0–98.3%, *FLOPs* by 90.4–98.4% and weight-file size by 95.1–99.1% at the respective storage precisions. Its accuracy is also higher, with gains of 6.5–9.3 percentage points in *mAP*@0.5 and 20.4–21.0 points in *mAP*@[0.5:0.95]. Compared with RT-DETR-18 [43], YOLOv7-tiny and YOLOv8n, the parameter and FLOP reductions are 21.3–88.1% and 25.9–89.5%, respectively.

However, YOLOv5n remains smaller: DSF-YOLO requires an additional 0.47 M parameters, 1.5 G *FLOPs* and 1.1 MB of weight storage. These additional costs yield gains of 1.7 and 4.4 points in the two *mAP* measures and 66.5 FPS in throughput relative to YOLOv5n; this exchange reflects the trade-off intended by the low-cost multi-scale design.

The comparison with recent YOLO models shows a similar balance between accuracy and cost. DSF-YOLO uses 8.14%, 7.06% and 4.44% fewer parameters than YOLO11n, YOLO12n and YOLO13n, respectively. Relative to the YOLO11n baseline, the two *mAP* measures improve by 1.4 and 4.9 points; relative to YOLO12n and YOLO13n, *mAP*@0.5 increases by 3.4 and 1.1 points, while reported throughput increases by 10.1% and 20.5%.

**Figure 11:**
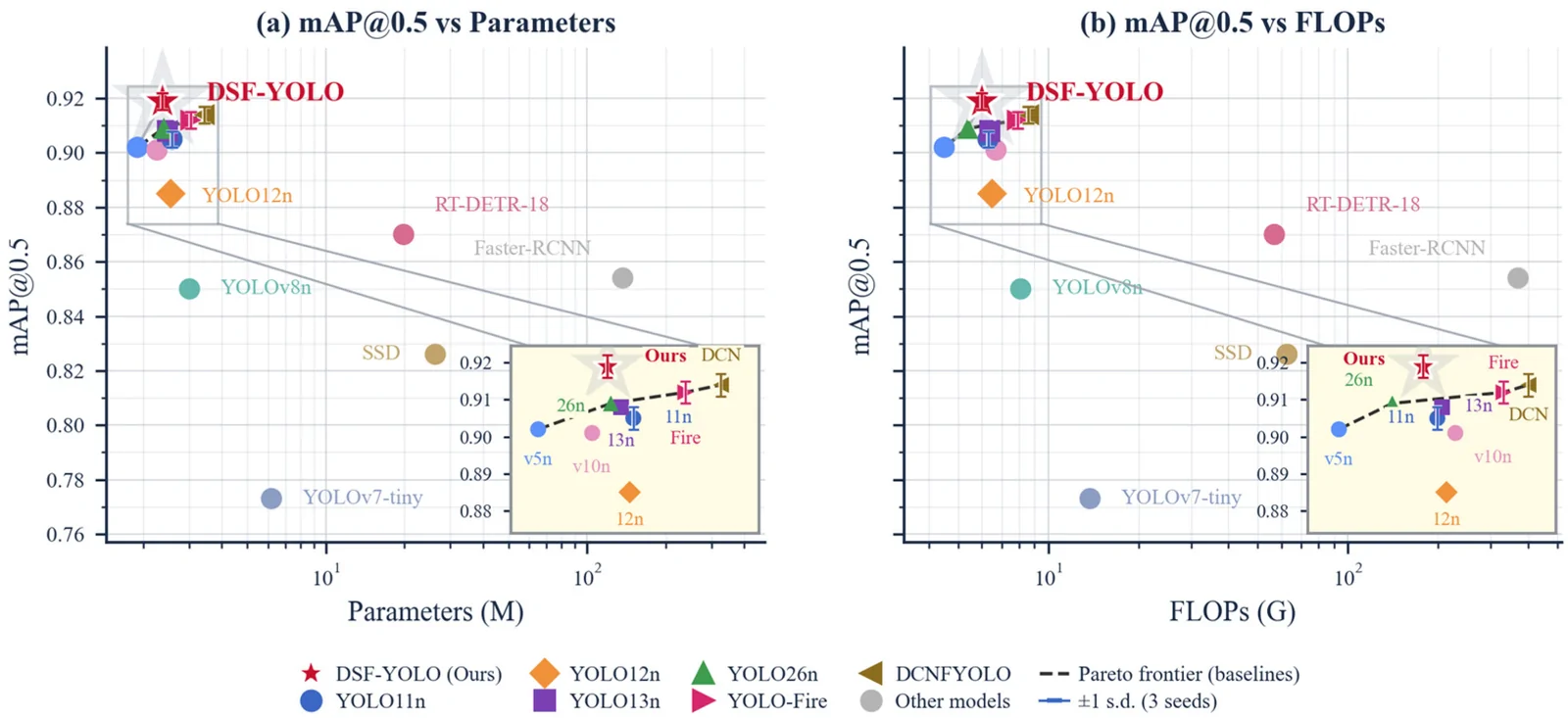
Accuracy–complexity comparison on DSF: (**a**) *mAP*@0.5 as a function of parameter count and (**b**) *mAP*@0.5 as a function of *FLOPs*, with logarithmic x-axes. DSF-YOLO is marked by a red star. The dashed line connects the generic YOLO baselines; the specialized detectors are plotted for reference.

The model requires 0.11 M more parameters than YOLOv10n but 0.7 G *FLOPs* less, so these two cost measures favor different models. Relative to YOLO26n, DSF-YOLO has 1.25% fewer parameters, 1.0 percentage point higher *mAP*@0.5 and 7.7% higher reported FPS; however, it requires 0.6 G *FLOPs* more and a 0.10 MB larger weight file. Overall, DSF-YOLO achieves the highest reported *mAP*@0.5 of 0.919 and *mAP*@[0.5:0.95] of 0.680 in Table 5. Its 175.0 FPS is the highest and its batch-1 latency of 7.2 ms the lowest among all compared models, and all margins of one point or more exceed the reported seed-level variability of ±0.003 in *mAP*@0.5; smaller margins are discussed below accordingly. FPS reflects sustained throughput at batch size 16, while latency denotes single-image inference; the two are reported separately and are not reciprocals. Figure 11 places these accuracy results alongside parameter count and *FLOPs*, making the cost differences visible without treating either complexity measure as a direct substitute for speed.

Table 5 also includes two detectors specialized for fire and smoke, retrained under the identical protocol with three random seeds. DCNFYOLO attains the highest per-class *AP* in Table 6 on large fire at 0.982 and on large smoke at 0.954, and its gains over YOLO11n decrease monotonically with class scale, from 1.7 points on large fire to 1.0, 0.6 and 0.3 points on large smoke, small fire and small smoke, respectively. DCNFYOLO was originally designed for single-class smoke detection [21]; its behavior under the four-class retraining therefore reflects the architecture operating under this protocol rather than under its original configuration. YOLO-Fire shows a different pattern consistent with its fire-domain design and P2 detection head: its gains over YOLO11n concentrate on the fire classes, 1.1 points on large fire and 0.9 points on small fire, while the smoke classes gain only 0.7 and 0.1 points. Neither profile reaches the small-scale regime where DSF-YOLO leads. On small fire, DSF-YOLO exceeds both specialized models by 1.6 to 1.9 points, reaching 0.921 compared with 0.905 for YOLO-Fire and 0.902 for DCNFYOLO; on small smoke it leads by 1.0 to 1.2 points, reaching 0.828 compared with 0.816 and 0.818. DSF-YOLO also leads both merged classes with 0.959 on fire and 0.912 on smoke, while the corresponding values for the specialized models are at most 0.957 and 0.910. In overall accuracy the three models are close: DSF-YOLO leads by 0.5 to 0.7 points in *mAP*@0.5, about 1.2 to 1.6 times the quadrature combination of the seed-level standard deviations, and by 2.2 to 2.6 points in *mAP*@[0.5:0.95]. It simultaneously uses 22.3 to 30.7% fewer parameters and reports both the highest throughput and the lowest batch-1 latency, at 175.0 FPS and 7.2 ms against 96.8 to 121.5 FPS and 9.9 to 12.1 ms for the two specialized models. DSF-YOLO is therefore ahead of its nearest competitors on every reported axis at once: both *mAP* measures, parameter count, throughput and latency. The distribution of gains, rather than the small overall margin, is the robust difference: the multi-scale design of DSF-YOLO, not fire-domain specialization, accounts for its advantage on small-scale targets.

**Figure 12:**
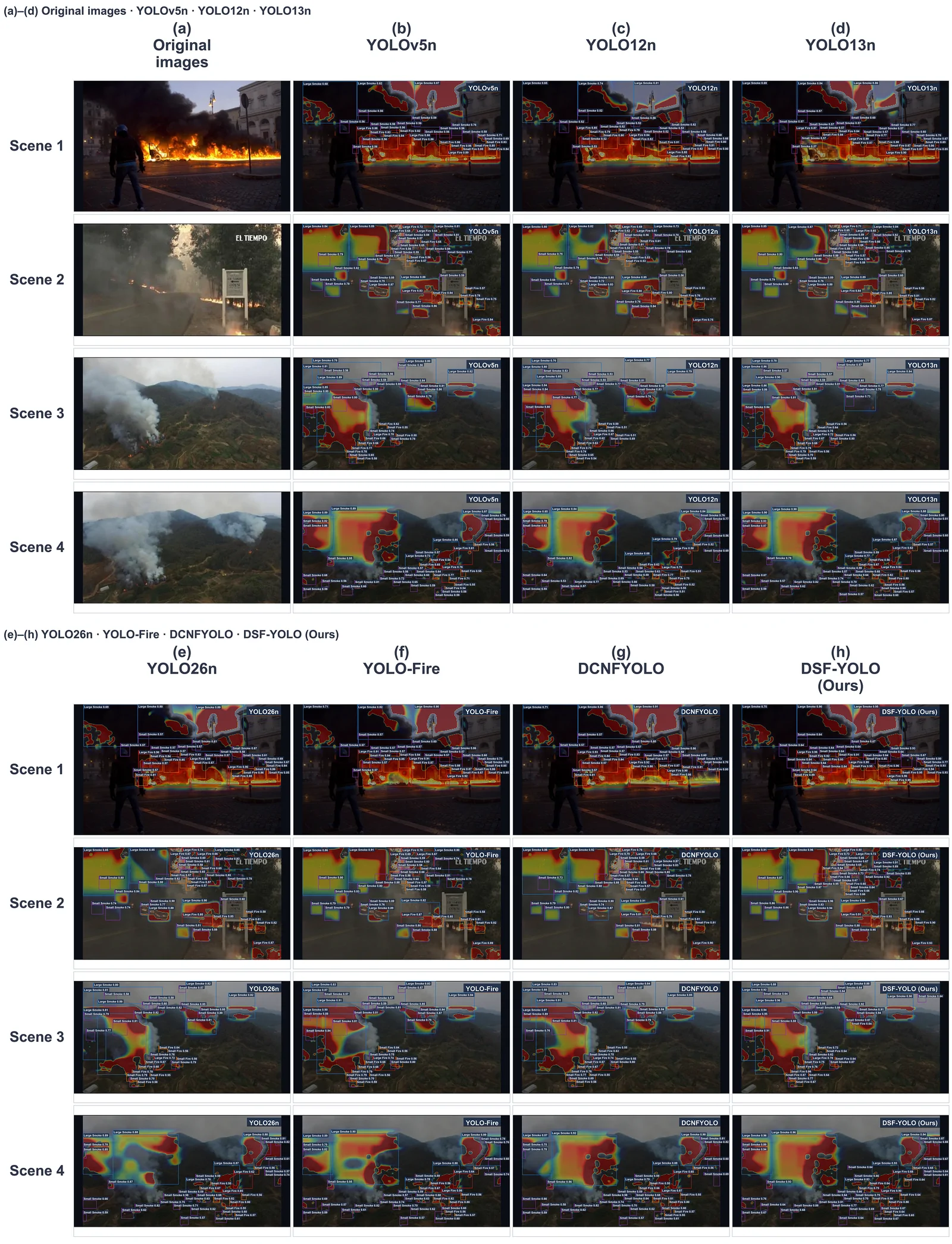
Heatmap analysis of the compared networks on the DSF dataset.

Figure 12 complements the numerical comparison with heatmaps and bounding-box overlays for four selected DSF images. The panels include the two fire- and smoke-specific detectors and allow highlighted regions and box coverage over visible fire, smoke and surrounding background to be compared across models on identical scenes. In the second scene, the YOLOv5n panel contains 36 boxes with displayed scores between 0.56 and 0.89, whereas the DSF-YOLO panel contains 40 boxes with scores between 0.64 and 0.96. In the fourth scene, DCNFYOLO contains 38 boxes with scores between 0.55 and 0.92, while DSF-YOLO contains 41 boxes, with small fire and small smoke labels reaching 0.90 and 0.94, respectively. YOLO26n remains competitive in Table 5 but labels one small fire target at 0.57, compared with 0.65 for the corresponding target in the DSF-YOLO panel. The additional DSF-YOLO boxes belong to the small-target classes, consistent with the per-class trends in Table 6. These examples illustrate scene-dependent differences between panels and accompany the aggregate accuracy results in Table 5.

The aggregate metrics alone do not show where these gains arise, yet object scale is the central design target of this work. Three evaluations at different granularities provide this evidence. Along the class axis in Table 6, DSF-YOLO improves AP over YOLO11n by 1.0 and 0.8 points on the large classes but by 2.5 and 1.3 points on the small classes. Along the COCO area axis in Table 3, which is independent of the class definition, the margins decrease monotonically with object size, at 6.0, 4.4 and 2.5 points for the small, medium and large bands. Under the merged two-class protocol, DSF-YOLO reaches 0.959 on fire and 0.912 on smoke, while YOLO11n reaches 0.944 and 0.902. The class and area axes agree that the improvement concentrates on smaller targets, the regime of weak local evidence and low contrast that motivates the multi-scale design, and the merged two-class protocol confirms that this conclusion does not depend on the four-class label definition. DSF-YOLO is also the smallest model within one percentage point of the best *mAP*@0.5 in Table 5, at 2.37 M parameters compared with 2.40 to 3.42 M for the other models in that group, and it leads every baseline in *mAP*@[0.5:0.95] by at least 1.5 points. The heatmap examples in Figure 12 complement these results qualitatively; quantifying background suppression would require targeted false-positive measurements.

#### 3.3.2. Comparison Experiment on the D-Fire Dataset

As introduced in Section 3.1, D-Fire provides an independent two-class fire and smoke benchmark; all models listed in Table 5 were retrained on its training set and evaluated on its test set under the identical protocol of Section 3.2. DSF-YOLO attains the highest accuracy on this benchmark, at 0.806 *mAP*@0.5 and 0.541 *mAP*@[0.5:0.95], with per-class *AP* of 0.831 on fire and 0.781 on smoke.

Relative to the YOLO11n baseline, the gains are 2.6 and 2.6 points in the two *mAP* measures; relative to the strongest specialized detector, YOLO-Fire at 0.800 and 0.534, the margins are 0.6 and 0.7 points, achieved with 22.3% fewer parameters. DCNFYOLO reaches 0.790 and 0.525 with 3.42 M parameters. The same three models again occupy the top three positions on both benchmarks, with DSF-YOLO first in each, which indicates that the advantage of the proposed architecture transfers to an independently curated fire benchmark. The per-class margins are consistent with the DSF observations: fire detection is more accurate than smoke detection for every model, and the gap for DSF-YOLO is 5.0 points, within the range of 4.0 to 8.0 points observed across all models.

The margin over the specialized detectors in *mAP*@0.5 is 0.6 to 1.6 points, two to more than five times the seed-level standard deviation reported in Table 7, and should be read as slight. The more robust differences are the margin in *mAP*@[0.5:0.95], at 0.7 to 1.6 points, and the cost advantage, at 22.3 to 30.7% fewer parameters and higher throughput than the two specialized models. This mirrors the pattern on DSF: the multi-scale design provides its clearest benefit in localization-sensitive metrics and model cost rather than in overall *mAP*@0.5 alone. Together with the results of Section 3.3.1, these findings support the claim that the proposed model performs robustly on independently curated fire benchmarks. The generic-detection references on VOC2012 and VisDrone2019 are reported below.

#### 3.3.3. Comparison Experiment on the VOC2012 Dataset and VisDrone2019 Dataset

To examine whether the proposed changes remain beneficial beyond the fire and smoke domain, Table 8 reports comparisons on the two non-fire benchmarks introduced in Section 3.1, which serve as a reference for transferable feature learning rather than as evidence of generalization across fire and smoke conditions.

On VOC2012, DSF-YOLO attains 0.695 *mAP*@0.5 and 0.535 *mAP*@[0.5:0.95], matching Faster R-CNN at 0.694 and 0.533 within a margin comparable to seed-level variability, while using approximately 1.7% and 1.6% of its parameters and *FLOPs*. Relative to the YOLO11n baseline, the two *mAP* measures improve by 5.7 and 5.8 percentage points. The model also records higher accuracy than YOLO12n, YOLO13n and YOLO26n on this benchmark.

On VisDrone2019, DSF-YOLO reaches 0.365 *mAP*@0.5 and 0.203 *mAP*@[0.5:0.95]. Relative to YOLO11n, the absolute gains are 1.5 and 1.6 percentage points, equivalent to relative increases of 4.3% and 8.6%. Compared with YOLO13n, the gains are 1.2 and 1.4 points; compared with YOLO26n, 0.7 and 1.2 points. The advantage thus persists under stricter localization requirements, consistent with the multi-scale design intent, although the two aggregate metrics alone do not separate localization, classification and missed detections. VisDrone2019 is the only benchmark in this study whose difficulty is dominated by small objects, and the persistence of the margin there is consistent with the scale concentration of the improvements on DSF.

### 3.4. Ablation Study

#### 3.4.1. Module-Level Ablation

Table 9 reports the sixteen combinations against the YOLO11n anchor of 0.905 *mAP*@0.5 and 0.631 *mAP*@[0.5:0.95]. ADown alone lowers the parameter count by 18.6%, *FLOPs* by 15.9% and the weight-file size by 17.3%, while *mAP*@0.5 decreases from 0.905 to 0.902, within one standard deviation, and *mAP*@[0.5:0.95] rises from 0.631 to 0.642; its isolated role is therefore cost reduction, and its accuracy effect depends on the surrounding modules. The larger parameter saving of ADown in isolation reflects the additional backbone transitions replaced in that configuration, whereas in the complete model ADown acts at the head transitions, where channel widths are narrower; the savings therefore depend on the replaced transitions and are not additive across configurations. DyFusion, C3K2-MFB and AIM-MSAIoU alone reach 0.913 and 0.657, 0.914 and 0.660, and 0.911 and 0.651, with gains of 0.8, 0.9 and 0.6 points on *mAP*@0.5 and 2.6, 2.9 and 2.0 points on *mAP*@[0.5:0.95] over the baseline. DyFusion and C3K2-MFB together reach 0.916 and 0.668, above either module alone.

**Figure 13:**
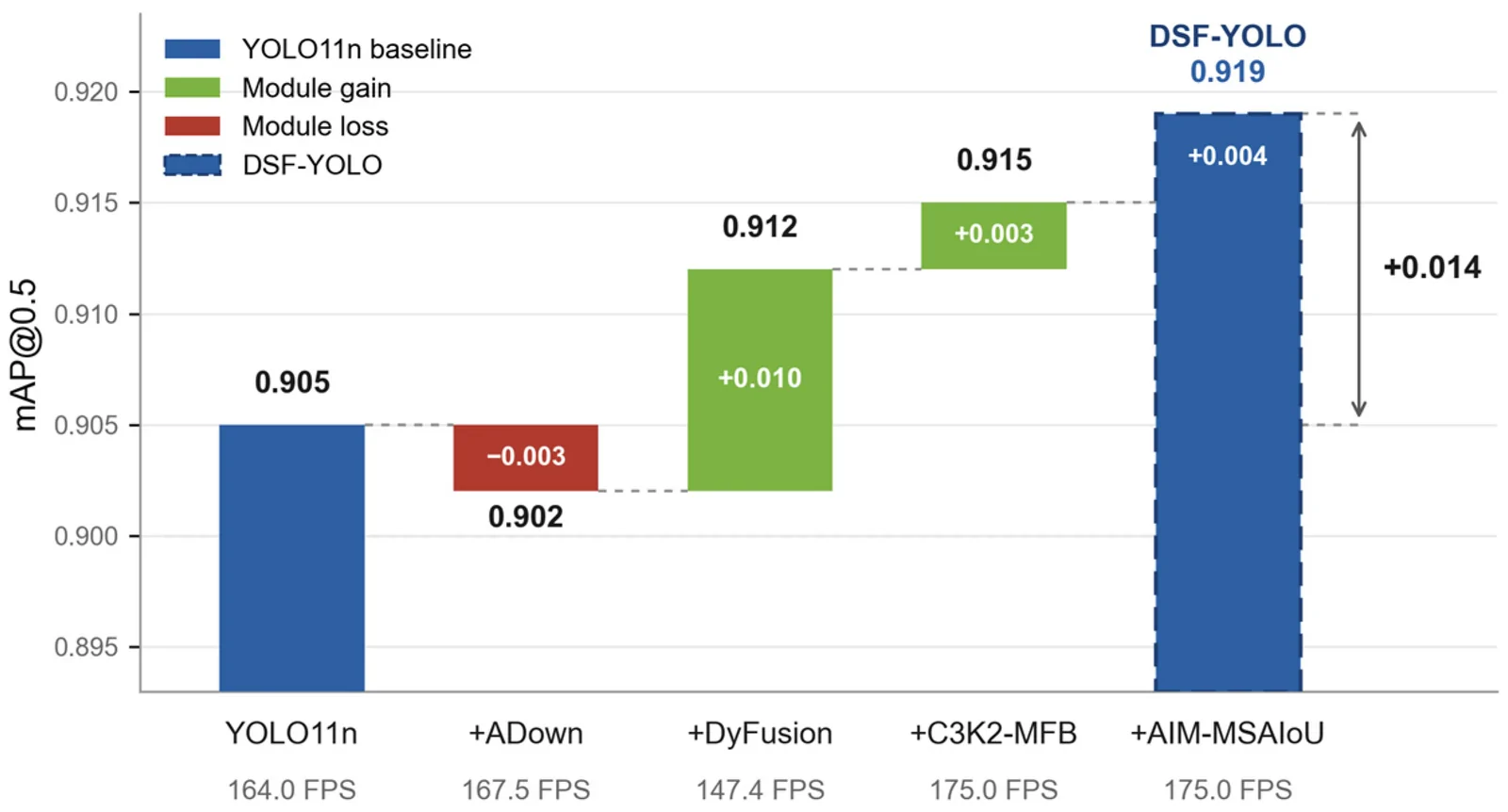
Cumulative ablation path from YOLO11n to DSF-YOLO. Each label adds one module to the preceding configuration; the bars show *mAP*@0.5 and the annotations report the measured throughput. Values correspond to the baseline, A, A + D, A + D + W and A + D + W + L rows of Table 9.

Figure 13 traces the cumulative path through A, A + D, A + D + W to the complete model, with scores of 0.902 and 0.642, 0.912 and 0.656, 0.915 and 0.674, and 0.919 and 0.680. The complete model attains the highest accuracy in Table 9 with 8.14% fewer parameters and 4.76% fewer *FLOPs* than YOLO11n. Relative to D + W + L, adding ADown retains this accuracy while reducing parameters from 2.51 M to 2.37 M and raising throughput from 149.9 to 175.0 FPS. Because AIM-MSAIoU acts only during training, configurations that differ solely in L share the same throughput, including A + D + W and the complete model at 175.0 FPS. The remaining throughput spread at similar *FLOPs*, most visibly the 68.3 FPS of A + W and A + W + L, reflects operator-level costs that *FLOPs* do not capture and is analyzed in Appendix C.

**Figure 14:**
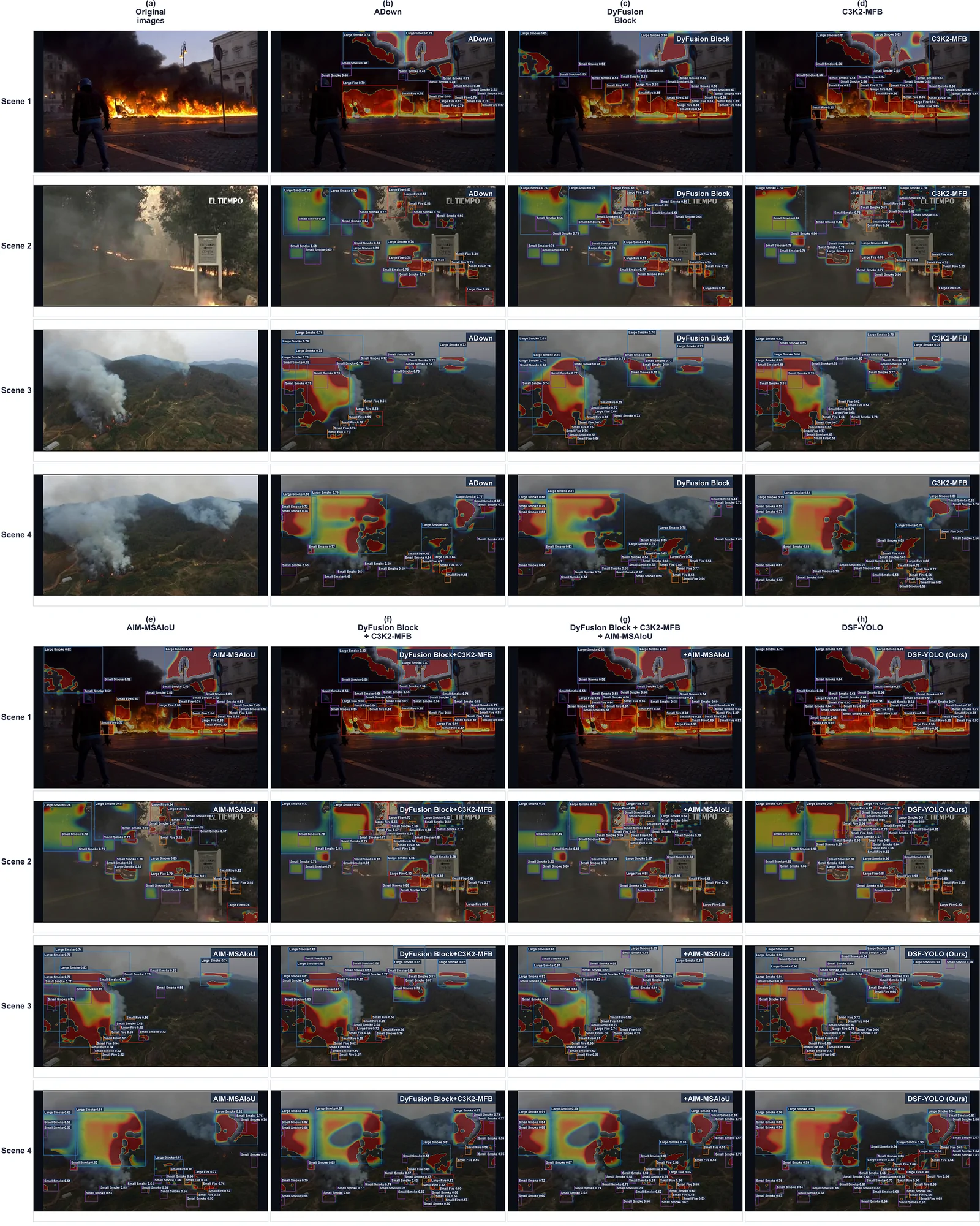
Selected ablation heatmaps on DSF. Each configuration is relative to YOLO11n.

Figure 14 shows heatmaps for the same scenes across selected configurations. Adding L to D + W changes mAP@0.5 from 0.916 to 0.918 and mAP@[0.5:0.95] from 0.668 to 0.677, and adding A brings the scores to 0.919 and 0.680. The mAP@0.5 increments are small relative to the reported seed-level standard deviations, while the larger mAP@[0.5:0.95] increase for L remains descriptive because seed-level variability is not reported for D + W + L. The panels provide a spatial comparison of these configurations. In the third scene, the ADown panel contains 20 boxes with displayed scores between 0.51 and 0.79, D + W + L contains 30 boxes with scores between 0.58 and 0.89, and the complete model contains 35 boxes with scores between 0.64 and 0.96. The corresponding small-target box counts are 14, 24 and 28, respectively. The heatmaps are read alongside the bounding-box overlays rather than as a direct measure of accuracy. In Table 9, the module gains are non-additive: D + W improves on either module alone but recovers about two thirds of the sum of their individual gains on both mAP measures. No single module matches the accuracy of the complete model, and the complete model is the only configuration that attains the highest accuracy in Table 9, together with fewer parameters, fewer FLOPs and higher throughput than YOLO11n.

#### 3.4.2. Intra-Module Design Choices

The intra-module analyzes isolate one design decision at a time on YOLO11n with a single module present, so each reference configuration is the corresponding single-module row of Table 9. Focal Loss [44], Quality Focal Loss [45] and SlideLoss [46] from the initial submission supervise classification rather than box geometry; their comparison is retained in Appendix B and excluded from the following controlled evaluations.

Table 10 varies only the regression term of an otherwise identical training objective. AIM-MSAIoU attains the highest accuracy at 0.911 and 0.651, a gain of 0.6 and 2.0 points over CIoU. The auxiliary box is the largest single contributor, reaching 0.909 and 0.650 alone through Inner-IoU; the corner term normalized by target dimensions follows at 0.906 and 0.642 and improves on the diagonal normalization of MPDIoU at 0.904 and 0.633, a result consistent with the large-scale variation of fire and smoke targets. Among the losses that do not decompose into these components, WIoU [47] reaches the highest values at 0.906 and 0.640, although the single-run differences among SIoU, EIoU and WIoU on *mAP*@0.5 lie within seed-level variability; all remain below the complete formulation. The components are not strictly additive: the auxiliary box combined with either corner formulation falls below Inner-IoU alone. The shape gain restores the complete formulation to 0.911 and 0.651, an advantage of 0.2 and 0.1 points over the strongest single component, which is comparable to the seed-level variability and is treated as indicative.

Accuracy peaks at α = 0.7 and varies within 0.4 and 0.6 points, as α ranges from 0.5 to 0.9; every sweep point remains above the CIoU baseline and above WIoU on both measures. This flat accuracy profile is consistent with the trade-off described in Section 2.4: a smaller ratio narrows the overlap range of the auxiliary boxes, whereas a ratio close to 1 reduces them to the plain IoU term, which is less sensitive at high overlap. The value α = 0.7 is therefore retained as a robust default rather than a finely tuned value. The remaining parameters are fixed at τ = 0.5, k = 10, a_2_ = 0.2 and a_1_ = 1.0 in all experiments; they were set once before the ablation campaign and were not tuned per configuration.

Table 11 varies the internal design of C3K2-MFB. A single decomposition level lowers the scores to 0.911 and 0.655, and a third level lowers them to 0.912 and 0.658 while raising *FLOPs* to 6.3 G and reducing throughput to 97.8 FPS, so two levels give the best balance. Removing the detail bands or the approximation band lowers the scores to 0.909 and 0.651 and to 0.910 and 0.653, so coarse structure and directional detail carry complementary information, with the directional detail components contributing slightly more. Removing SE changes *mAP*@0.5 by 0.2 points at unchanged cost, a difference within seed-level variability. Replacing the wavelet stages with dilated convolutions at a matched parameter budget reaches 0.910 and 0.654, 0.4 and 0.6 points below the default. The dilated-convolution replacement and the approximation-only variant are indistinguishable on *mAP*@0.5 at 0.910 and 0.909 in single runs; the advantage of the default over both variants is carried mainly by the *mAP*@[0.5:0.95] margins, 0.660 against 0.654 and 0.651, and is treated as tentative. The replacement is faster, at 117.3 FPS compared with 104.5 FPS, and this gap is recovered at the system level by ADown. A further alternative would be heavier cross-stage fusion in the neck, but YOLO11 already provides a feature pyramid there, whereas C3K2-MFB acts within single-stage feature extraction; the two are positioned as complementary rather than interchangeable, although their combination is not tested in this study.

Table 12 places SE and DyFusion at the same positions on YOLO11n. SE raises the baseline to 0.908 and 0.636 at 158.6 FPS, whereas DyFusion reaches 0.913 and 0.657 at 141.7 FPS; the contextual path thus adds 0.5 and 2.1 points over the lightweight alternative at an additional cost of 16.9 FPS, and ADown recovers this throughput along the cumulative path of Section 3.4.1.

## 4. Conclusions

This study presents DSF-YOLO for fire and smoke detection with an emphasis on accuracy and computational cost. The model combines wavelet-based C3K2-MFB with local and contextual feature processing in DyFusion Block, while ADown reduces the cost of downsampling. AIM-MSAIoU further provides box-regression supervision through fixed-scale auxiliary-box overlap and a shape-weighted corner penalty normalized by target size. These components address different parts of the detection process, and their combined effect is evaluated on the 7572-image DSF dataset. Relative to YOLO11n, the full model increases *mAP*@0.5 from 0.905 to 0.919 and *mAP*@[0.5:0.95] from 0.631 to 0.680, corresponding to gains of 1.4 and 4.9 percentage points. Parameter count and *FLOPs* decrease by 8.14% and 4.76%, respectively, and weight-file size decreases from 5.20 to 4.90 MB. Throughput rises from 164.0 to 175.0 FPS at a batch size of 16 on the same GPU. Overall, these measurements indicate that the full model improves the reported accuracy–cost balance over the baseline; the ablation results also show that individual component effects depend on the surrounding configuration.

DSF-YOLO also attains the highest accuracy among the compared detectors on the D-Fire benchmark and on VisDrone2019, and matches the strongest baseline on VOC2012, Faster R-CNN, at approximately 1.7% of its parameters and 1.6% of its *FLOPs*. These additional evaluations suggest that the architecture is useful beyond the DSF setting, but they do not establish robustness to every fire-monitoring condition. Further work should extend the size-stratified analysis to additional independent fire benchmarks, examine robustness under varying illumination and weather conditions, and evaluate latency, memory use and power consumption on embedded hardware. Such tests would clarify whether the observed accuracy and throughput translate into reliable operation on mobile or embedded platforms.

## 5. Patents

Potential applications of DSF-YOLO include continuous video-based fire and smoke monitoring in forest, urban and indoor scenarios, in particular on platforms with limited computational resources.

## Figures and Tables

**Table 1:** Composition of the Dual-Scale Fire (DSF) dataset: number of images and instances per class in the training, validation, and test subsets under the source-grouped 8:1:1 split.

Class	Train Images	Val Images	Test Images	Train Instances	Val Instances	Test Instances	Total Instances
LF	2341	293	292	7152	894	894	8940
LS	2005	251	250	5772	722	721	7215
SF	1648	206	205	3506	438	438	4382
SS	1522	190	189	2889	361	361	3611
Total	6057	757	758	19,319	2415	2414	24,148

An image may contain instances of multiple classes; class-wise image counts therefore sum to more than the number of unique images. Instances are counted per bounding box.

**Table 2:** Sensitivity of the class definition to the size threshold on the DSF test set.

Threshold	Small Instances	Large Instances	Small Share	*mAP*@0.5	*mAP*@[0.5:0.95]
0.5%	486	1928	20.1%	0.917	0.675
1.0%	799	1615	33.1%	0.919	0.680
2.0%	1214	1200	50.3%	0.920	0.681

The model is not retrained; ground-truth and predicted labels are re-merged by the same area-threshold rule before evaluation, so instance counts vary monotonically with the threshold.

**Table 3:** Size-stratified detection accuracy on the DSF test set, computed with the COCO area bands.

Size Band	GT Instances	YOLO11n *AP*@[0.5:0.95]	DSF-YOLO *AP*@[0.5:0.95]	Δ (pp)
Small (<32^2^)	612	0.460	0.520	6.0
Medium (32^2^–96^2^)	1489	0.650	0.694	4.4
Large (≥96^2^)	313	0.880	0.905	2.5
All	2414	0.631	0.680	4.9

Areas are computed on the letterboxed 640 × 640 input, independent of the 1% threshold; the All row equals the overall mAP@[0.5:0.95], not an average of the bands.

**Table 4:** Environment configuration.

Name	Configuration
System	Ubuntu 22.04 LTS (Canonical Ltd., London, UK)
Environment	Python-3.10 + torch-2.1.2 + CUDA-12.4
CPU	18 vCPU AMD EPYC 9754 (Advanced Micro Devices, Inc., Santa Clara, CA, USA)
GPU	NVIDIA GeForce RTX 4090D (24 GB) (NVIDIA Corporation, Santa Clara, CA, USA)
Numerical precision	FP32 (training and timing)
Timing protocol	batch 16 and batch 1; 50 warm-up + 200 timed iterations; torch.cuda.synchronize() before and after timing

**Table 5:** Comparison results on the DSF dataset: overall accuracy, model cost and throughput.

Model	Para/M	*FLOPs*/G	*mAP*@0.5	*mAP*@[0.5:0.95]	Size/MB	FPS	Latency/ms
Faster R-CNN	137.09	370.2	0.854	0.476	522.99	43.8	26.8
SSD	26.28	62.7	0.826	0.470	100.27	74.9	14.4
RT-DETR-18	19.87	56.9	0.870	0.665	76.90	79.7	13.0
YOLOv5n	1.90	4.5	0.902	0.636	3.80	108.5	10.3
YOLOv7-tiny	6.20	13.8	0.773	0.438	12.40	156.5	7.9
YOLOv8n	3.01	8.1	0.850	0.566	6.00	108.4	10.4
YOLOv10n	2.26	6.7	0.901	0.665	4.50	80.1	12.9
YOLO11n	2.58	6.3	0.905 ± 0.003 ^b^	0.631 ± 0.004 ^b^	5.20	164.0 ± 0.9 ^b^	7.4
YOLO12n	2.55	6.5	0.885	0.623	5.10	159.0	7.7
YOLO13n	2.48	6.4	0.908	0.642	5.00	145.2	8.3
YOLO26n	2.40	5.4	0.909	0.645	4.80	162.5	7.6
DSF-YOLO (ours)	2.37	6.0	0.919 ± 0.003 ^b^	0.680 ± 0.004 ^b^	4.90	175.0 ± 0.7 ^b^	7.2
YOLO-Fire [17] ^c^	3.05	7.9	0.912 ± 0.003 ^b^	0.654 ± 0.005 ^b^	6.20	121.5 ± 1.0 ^b^	9.9
DCNFYOLO [21] ^c^	3.42	8.6	0.914 ± 0.003 ^b^	0.658 ± 0.004 ^b^	6.90	96.8 ± 0.9 ^b^	12.1

^b^ Mean ± sample standard deviation over three random seeds; the standard deviation reflects training stochasticity on a fixed test set and does not include test-resampling variability. For FPS, the standard deviation is taken over the timing runs of the three seed checkpoints and reflects measurement variability rather than training stochasticity, since throughput is dominated by the deployed architecture. Model sizes are the released checkpoint file sizes; the Ultralytics checkpoints are stored in half precision at approximately two bytes per parameter, whereas Faster R-CNN, SSD and RT-DETR-18 checkpoints are stored in FP32, so size comparisons across families reflect storage precision as well as parameter count. ^c^ Fire- and smoke-specific detectors retrained under the identical protocol. The *FLOPs* of Faster R-CNN and SSD are commonly cited literature values obtained under their native configurations, with each multiply–accumulate operation counted twice; the remaining models were profiled with thop at 640 × 640, where multiply–accumulate operations are counted once. All latency and FPS measurements were conducted at 640 × 640.

**Table 6:** Per-class AP@0.5 and merged two-class AP on the DSF test set.

Model	AP_LF	AP_LS	AP_SF	AP_SS	AP_Fire ^d^	AP_Smoke
Faster R-CNN	0.914	0.888	0.845	0.769	0.893	0.850
SSD	0.886	0.860	0.818	0.740	0.865	0.821
RT-DETR-18	0.930	0.905	0.863	0.782	0.909	0.865
YOLOv5n	0.960	0.938	0.898	0.811	0.941	0.897
YOLOv7-tiny	0.837	0.811	0.766	0.678	0.815	0.768
YOLOv8n	0.909	0.886	0.843	0.762	0.889	0.846
YOLOv10n	0.960	0.937	0.899	0.808	0.941	0.895
YOLO11n	0.965	0.944	0.896	0.815	0.944	0.902
YOLO12n	0.944	0.921	0.880	0.795	0.924	0.880
YOLO13n	0.966	0.945	0.904	0.817	0.947	0.904
YOLO26n	0.969	0.945	0.903	0.819	0.949	0.904
DSF-YOLO (ours)	0.975	0.952	0.921	0.828	0.959	0.912
YOLO-Fire [17] ^c^	0.976	0.951	0.905	0.816	0.954	0.907
DCNFYOLO [21] ^c^	0.982	0.954	0.902	0.818	0.957	0.910

^c^ Fire- and smoke-specific detectors. ^d^ Merged two-class results: the four classes are remapped to fire (LF + SF) and smoke (LS + SS) with class-agnostic re-NMS before evaluation. LF/LS/SF/SS denote large fire, large smoke, small fire and small smoke, respectively. Per-class precision and recall at a fixed confidence of 0.25 are provided in Appendix B, Table A3.

**Table 7:** Comparison results on the D-Fire dataset under the two-class fire and smoke taxonomy. All models are retrained on the D-Fire training set and evaluated on its test set under the identical protocol of Section 3.2.

Model	*mAP*@0.5	*mAP*@[0.5:0.95]	AP_Fire	AP_Smoke
Faster R-CNN	0.660	0.420	0.700	0.620
SSD	0.700	0.448	0.730	0.670
RT-DETR-18	0.776	0.507	0.802	0.750
YOLOv5n	0.770	0.506	0.795	0.745
YOLOv7-tiny	0.785	0.512	0.815	0.755
YOLOv8n	0.778	0.511	0.803	0.753
YOLOv10n	0.772	0.510	0.797	0.747
YOLO11n	0.780 ± 0.003 ^b^	0.515 ± 0.004 ^b^	0.805	0.755
YOLO12n	0.775	0.512	0.800	0.750
YOLO13n	0.782	0.517	0.807	0.757
YOLO26n	0.784	0.518	0.804	0.764
DSF-YOLO (ours)	0.806 ± 0.003 ^b^	0.541 ± 0.003 ^b^	0.831	0.781
YOLO-Fire [17] ^c^	0.800 ± 0.003 ^b^	0.534 ± 0.005 ^b^	0.830	0.770
DCNFYOLO [21] ^c^	0.790 ± 0.003 ^b^	0.525 ± 0.003 ^b^	0.815	0.765

^b^ Mean ± standard deviation over three random seeds. ^c^ Fire- and smoke-specific detectors. Model cost and throughput are architecture-level quantities identical across evaluation datasets and are reported once in Table 5. AP_fire and AP_smoke denote per-class AP@0.5 under the native two-class taxonomy of D-Fire.

**Table 8:** Comparison results on VOC2012 and VisDrone2019.

Model	VOC *mAP*@0.5	VOC *mAP*@[0.5:0.95]	VisDrone *mAP*@0.5	VisDrone *mAP*@[0.5:0.95]
Faster R-CNN	0.694	0.533	0.212	0.118
SSD	0.622	0.463	0.237	0.129
RT-DETR-18	0.654	0.479	0.250	0.135
YOLOv5n	0.580	0.400	0.263	0.150
YOLOv7-tiny	0.579	0.404	0.246	0.159
YOLOv8n	0.588	0.409	0.298	0.163
YOLOv10n	0.589	0.431	0.275	0.159
YOLO11n	0.638	0.477	0.350	0.187
YOLO12n	0.643	0.483	0.321	0.190
YOLO13n	0.641	0.479	0.353	0.189
YOLO26n	0.640	0.470	0.358	0.191
DSF-YOLO (ours)	0.695	0.535	0.365	0.203

Model cost and throughput are architecture-level quantities identical across evaluation datasets and are reported once in Table 5. VOC2012 results are based on the authors’ re-split of the annotated trainval set and are not comparable with official server-based test evaluations.

**Table 9:** Results of the module-level ablation study on the DSF dataset.

ADown	DyFusion Block	C3K2-MFB	AIM-MSAIoU	Para/M	*FLOPs*/G	*mAP*@0.5	*mAP*@[0.5:0.95]	Model Size/MB	FPS
×	×	×	×	2.58	6.3	0.905 ± 0.003 ^b^	0.631 ± 0.004 ^b^	5.2	164.0 ± 0.9 ^b^
√	×	×	×	2.10	5.3	0.902 ± 0.003 ^b^	0.642 ± 0.004 ^b^	4.3	167.5 ± 0.9 ^b^
×	√	×	×	2.46	6.2	0.913 ± 0.003 ^b^	0.657 ± 0.004 ^b^	5.0	141.7 ± 1.0 ^b^
×	×	√	×	2.52	6.2	0.914 ± 0.003 ^b^	0.660 ± 0.005 ^b^	5.1	104.5 ± 0.8 ^b^
×	×	×	√	2.58	6.3	0.911 ± 0.002 ^b^	0.651 ± 0.003 ^b^	5.2	164.0 ± 0.9 ^b^
√	√	×	×	2.33	6.0	0.912	0.656	4.8	147.4
√	×	√	×	2.40	6.1	0.913	0.659	4.9	68.3
√	×	×	√	2.10	5.3	0.906	0.650	4.3	167.5
×	√	√	×	2.51	6.2	0.916 ± 0.003 ^b^	0.668 ± 0.004 ^b^	5.1	149.9 ± 0.9 ^b^
×	√	×	√	2.46	6.2	0.915	0.664	5.0	141.7
×	×	√	√	2.52	6.2	0.916	0.667	5.1	104.5
√	√	√	×	2.37	6.0	0.915 ± 0.003 ^b^	0.674 ± 0.005 ^b^	4.9	175.0 ± 0.7 ^b^
√	√	×	√	2.33	6.0	0.916	0.672	4.8	147.4
√	×	√	√	2.40	6.1	0.917	0.675	4.9	68.3
×	√	√	√	2.51	6.2	0.918	0.677	5.1	149.9
√	√	√	√	2.37	6.0	0.919 ± 0.003 ^b^	0.680 ± 0.004 ^b^	4.9	175.0 ± 0.7 ^b^

√ indicates that the module is applied in the corresponding configuration; × indicates that it is not applied. ^b^ Mean ± standard deviation over three random seeds; remaining rows are single runs. A, D, W and L denote ADown, DyFusion Block, C3K2-MFB and AIM-MSAIoU. AIM-MSAIoU affects training only, so configurations that differ solely in L share the same deployed architecture and throughput.

**Table 10:** Controlled evaluation of the box-regression objective on YOLO11n; only the regression term of an otherwise identical training objective is varied. Decomposition rows isolate the three components, sensitivity rows vary α, and the row at α = 0.7 corresponds to the L row of Table 9.

Regression Term	Aux Box	Corner Term	Shape Gain	α	*mAP*@0.5	*mAP*@[0.5:0.95]
CIoU ^b^	×	×	×	—	0.905 ± 0.003	0.631 ± 0.004
SIoU	—	—	—	—	0.903	0.635
EIoU	—	—	—	—	0.905	0.638
WIoU	—	—	—	—	0.906	0.640
MPDIoU	×	√	×	—	0.904	0.633
MSAIoU	×	√	×	—	0.906	0.642
Inner-IoU	√	×	×	0.7	0.909	0.650
Inner-MPDIoU	√	√	×	0.7	0.905	0.644
Inner + MSAIoU	√	√	×	0.7	0.907	0.647
AIM-MSAIoU ^b^	√	√	√	0.7	0.911 ± 0.002	0.651 ± 0.003
AIM-MSAIoU, α = 0.5	√	√	√	0.5	0.907	0.645
AIM-MSAIoU, α = 0.6	√	√	√	0.6	0.910	0.649
AIM-MSAIoU, α = 0.8	√	√	√	0.8	0.910	0.650
AIM-MSAIoU, α = 0.9	√	√	√	0.9	0.908	0.647

^b^ Mean ± standard deviation over three random seeds; CIoU corresponds to the YOLO11n baseline and AIM-MSAIoU to the L row of Table 9; other rows are single runs. √ indicates that the component is present and × that it is absent; — indicates not applicable, since the corresponding loss does not decompose into these components. MPDIoU normalizes corner distances by the squared image diagonal, whereas MSAIoU normalizes them by the target width and height.

**Table 11:** Internal design ablation of C3K2-MFB on YOLO11n; the default row corresponds to the W row of Table 9. The dilated-convolution row replaces wavelet decomposition and inverse reconstruction with dilated convolutions at a matched parameter budget.

Configuration	Levels	Subbands	SE	Para/M	*FLOPs*/G	*mAP*@0.5	*mAP*@[0.5:0.95]	FPS
Default	2	*LL + LH + HL + HH*	√	2.52	6.2	0.914	0.660	104.5
Level 1	1	*LL + LH + HL + HH*	√	2.51	6.1	0.911	0.655	112.4
Level 3	3	*LL + LH + HL + HH*	√	2.53	6.3	0.912	0.658	97.8
Approximation only	2	*LL*	√	2.50	6.1	0.909	0.651	111.6
Detail only	2	*LH + HL + HH*	√	2.51	6.1	0.910	0.653	110.2
Without SE	2	*LL + LH + HL + HH*	×	2.52	6.2	0.912	0.658	105.1
Dilated convolution	—	—	—	2.52	6.2	0.910	0.654	117.3

√ indicates that the component (or term) is enabled; × indicates that it is disabled; — indicates that the item is not applicable to this configuration.

**Table 12:** Attention formulations placed at the DyFusion positions on YOLO11n.

Configuration	Para/M	*FLOPs*/G	*mAP*@0.5	*mAP*@[0.5:0.95]	FPS
YOLO11n	2.58	6.3	0.905	0.631	164.0
SE at the DyFusion positions	2.44	6.2	0.908	0.636	158.6
DyFusion Block	2.46	6.2	0.913	0.657	141.7

## Data Availability

The DSF dataset constructed in this study is available from the corresponding author upon reasonable request. It was compiled from publicly available imagery; in particular, the Open Wildfire Smoke Dataset was obtained from AI for Mankind and HPWREN, available online: https://github.com/aiformankind/wildfire-smoke-dataset (accessed on 4 September 2026). The bounding-box annotations of this dataset are distributed under the Creative Commons Attribution-NonCommercial-ShareAlike 4.0 (CC BY-NC-SA 4.0) license and were used for non-commercial academic research in accordance with its terms.

## Abbreviations

The following abbreviations are used in this manuscript:

ADown	Downsampling module (adopted from YOLOv9)
AIM-MSAIoU	Adaptive Inner-enhanced Multi-Scale Alignment intersection over union
C2PSA	Cross-stage partial with spatial attention
C3K2-MFB	Multi-frequency band C3K2 module
CBS	Convolution, batch normalization and SiLU
CGLU	Convolutional gated linear unit
CIoU	Complete intersection over union
DSF	Dual-Scale Fire
FLOPs	Floating-point operations
FPS	Frames per second
GDT-Block	Global Dependency Transformer Block
GELU	Gaussian error linear unit
GFLOPs	Giga floating-point operations
HPWREN	High-Performance Wireless Research and Education Network
Inner-IoU	Inner-region intersection over union
IoU	Intersection over union
LFE-Block	Local Feature Extraction Block
mAP	Mean average precision
MHSA	Multi-head self-attention
MPDIoU	Minimum point distance intersection over union
MSAIoU	Multi-Scale Alignment intersection over union
SE	Squeeze-and-excitation
SiLU	Sigmoid linear unit
YOLO	You Only Look Once

## Appendix A

Table A1 compares the source-grouped split adopted in this work with the random split of the same 8:1:1 ratio used in the initial submission. Under the grouped split, images sharing an HPWREN camera sequence, fire event or originating dataset are confined to a single subset, so that the training and test scenes are independent. The grouped split lowers *mAP*@0.5 by 0.4 points for YOLO11n and DSF-YOLO alike, and *mAP*@[0.5:0.95] by 0.4 and 0.5 points; these changes are comparable to the seed-level variability of Table 9. The reported accuracy therefore does not rely on source-level correlation between the training and test scenes, and the grouped-split values are adopted throughout the manuscript.

**Table A1:** Comparison of the random split and the source-grouped split on the DSF dataset under the unified protocol of Section 3.2.

Configuration	Split	*mAP*@0.5	*mAP*@[0.5:0.95]
YOLO11n	Random	0.909	0.635
YOLO11n	Source-grouped	0.905	0.631
DSF-YOLO	Random	0.923	0.685
DSF-YOLO	Source-grouped	0.919	0.680

## Appendix B

As noted in Section 3.4.2, the classification-side objectives of the initial submission are not interchangeable with a box-regression term and are therefore excluded from the controlled comparison. For completeness, Table A2 retains the corresponding YOLO11n training-loss configurations as a reference: these runs replace the complete training objective rather than the regression term alone. The Focal Loss and Quality Focal Loss configurations fall below the CIoU baseline. SlideLoss [46] reaches 0.891 in *mAP*@0.5, below the regression-side results of Table 10, whose *mAP*@0.5 values are at least 0.903, although its mAP@[0.5:0.95] of 0.641 is comparable to several rows of that table.

**Table A2:** YOLO11n training-loss configurations on the DSF dataset, reported in the initial submission and retained as a reference. These configurations replace the complete training objective and are not component-wise decompositions of the regression term. ^b^ Mean ± sample standard deviation over three random seeds; the remaining rows are single runs.

Training Configuration	*mAP*@0.5	*mAP*@[0.5:0.95]
CIoU baseline ^b^	0.905 ± 0.003	0.631 ± 0.004
Focal Loss	0.857	0.625
Quality Focal Loss	0.860	0.618
SlideLoss	0.891	0.641

Table A3 reports per-class precision and recall on the DSF test set at the fixed confidence threshold of 0.25 specified in Section 3.2, complementing the per-class AP of Table 6 with point estimates of the precision-recall trade-off. The macro-averaged precision, recall and F1 are additionally reported as unweighted means over the four classes. DSF-YOLO attains the highest macro-averaged precision at 0.845 and the highest macro-averaged F1 at 0.848, and its recall on small fire reaches 0.879 against 0.811 for YOLO11n, consistent with the scale concentration of the improvements reported in Section 3.3.1.

**Table A3:** Per-class precision (P) and recall (R) on the DSF test set at a fixed confidence threshold of 0.25.

Model	P_LF	R_LF	P_LS	R_LS	P_SF	R_SF	P_SS	R_SS	Macro-P	Macro-R	Macro-F1	mAP@0.5
Faster R-CNN	0.714	0.917	0.756	0.842	0.704	0.842	0.710	0.670	0.721	0.818	0.764	0.854
SSD	0.743	0.886	0.745	0.831	0.675	0.820	0.735	0.537	0.724	0.768	0.739	0.826
RT-DETR-18	0.829	0.885	0.771	0.874	0.745	0.826	0.661	0.762	0.752	0.837	0.792	0.870
YOLOv5n	0.832	0.944	0.733	0.956	0.836	0.804	0.730	0.726	0.783	0.857	0.815	0.902
YOLOv7-tiny	0.631	0.885	0.700	0.779	0.703	0.712	0.594	0.720	0.657	0.774	0.708	0.773
YOLOv8n	0.751	0.893	0.743	0.868	0.701	0.820	0.796	0.540	0.748	0.780	0.754	0.850
YOLOv10n	0.741	0.969	0.794	0.925	0.853	0.728	0.764	0.698	0.788	0.830	0.802	0.901
YOLO11n ^b^	0.818	0.954	0.841	0.910	0.818	0.811	0.770	0.687	0.812	0.840	0.824	0.905
YOLO12n	0.746	0.971	0.732	0.926	0.822	0.813	0.718	0.690	0.754	0.850	0.796	0.885
YOLO13n	0.894	0.914	0.804	0.917	0.800	0.831	0.779	0.673	0.819	0.834	0.824	0.908
YOLO26n	0.719	0.985	0.734	0.950	0.807	0.852	0.795	0.676	0.764	0.866	0.805	0.909
DSF-YOLO ^b^	0.894	0.943	0.883	0.904	0.816	0.879	0.787	0.687	0.845	0.853	0.848	0.919
YOLO-Fire ^c^	0.761	0.983	0.860	0.908	0.769	0.852	0.788	0.618	0.794	0.840	0.811	0.912
DCNFYOLO ^c^	0.835	0.982	0.819	0.945	0.762	0.897	0.829	0.524	0.811	0.837	0.812	0.914

^b^ Mean over three random seeds. ^c^ Fire- and smoke-specific detectors. LF, LS, SF and SS denote large fire, large smoke, small fire and small smoke. Precision and recall are computed at the fixed confidence threshold of 0.25 stated in Section 3.2. Macro-P, Macro-R and Macro-F1 are unweighted means over the four classes. The mAP@0.5 column reproduces Table 5 for cross-reference.

## Appendix C

Section 3.2 notes that *FLOPs* count multiply–accumulate operations of the forward pass only and do not capture memory traffic or kernel-launch overhead. Table A4 reports the per-stage latency share of four representative configurations, measured with torch.profiler under the timing protocol of Section 3.2 at a batch size of 1. Shares are expressed as percentages of the total batch-1 latency of each configuration. Batch-1 profiling is used because kernel-launch and memory-traffic overheads, which *FLOPs* do not capture, are most visible at small batch sizes; the FPS values discussed below are batch-16 throughput from Table 9, and the stage attribution is read as identifying which operations limit inference in both regimes.

**Table A4:** Per-stage latency share of representative configurations under the timing protocol of Section 3.2, measured with torch.profiler at a batch size of 1. Shares sum to 100% within each column; — indicates that the stage is absent from the configuration.

Stage	YOLO11n	A + W	DyFusion	DSF-YOLO
Convolution	76.8%	38.2%	62.9%	46.9%
Wavelet transform and reconstruction	—	34.6%	—	21.3%
DyFusion attention path	—	—	16.8%	17.6%
Downsampling	12.4%	18.3%	11.2%	5.4%
Letterbox and NMS	10.8%	8.9%	9.1%	8.8%

Three observations follow from Table A4. First, A + W and A + W + L report 68.3 FPS although their *FLOPs* are lower than those of YOLO11n; the wavelet transform and reconstruction, together with downsampling, account for 52.9% of the total latency in A + W, and these stages are memory-bound and kernel-launch intensive, so their cost is not reflected in the *FLOPs* count. In A + W, the downsampling share rises to 18.3% instead of falling, because the two branch ADown kernels feed the wavelet stages through split channels; the two memory-bound families therefore limit throughput jointly. Second, replacing the wavelet stages with dilated convolutions raises throughput from 104.5 to 117.3 FPS at a matched parameter budget and matched *FLOPs*, which isolate the memory traffic of the frequency transform, rather than the amount of arithmetic, as the cause of the gap. Third, the DyFusion attention path adds a 17.6% latency share, only partly offset by ADown lowering the downsampling share from 12.4% to 5.4%; the convolution share, falling from 76.8% to 46.9%, carries the remainder, so the batch-1 latency decreases from 7.4 to 7.2 ms and the complete model reports 175.0 FPS.
